# The RNA-binding protein CSDE1 promotes hematopoietic stem and progenitor cell generation via translational control of Wnt signaling

**DOI:** 10.1242/dev.201890

**Published:** 2023-10-24

**Authors:** Ying Li, Can Li, Mengyao Liu, Shicheng Liu, Feng Liu, Lu Wang

**Affiliations:** ^1^ State Key Laboratory of Experimental Hematology, National Clinical Research Center for Blood Diseases, Haihe Laboratory of Cell Ecosystem, Institute of Hematology and Blood Diseases Hospital, Chinese Academy of Medical Sciences and Peking Union Medical College, Tianjin 300020, China; ^2^ State Key Laboratory of Membrane Biology, Institute of Zoology, Institute for Stem Cell and Regeneration, Chinese Academy of Sciences, University of Chinese Academy of Sciences, Beijing 100101, China; ^3^ Tianjin Institutes of Health Science, Tianjin 301600, China

**Keywords:** Hematopoietic stem and progenitor cell, Post-transcriptional regulation, RNA-binding protein, Csde1, Wnt signaling, Zebrafish

## Abstract

In vertebrates, the earliest hematopoietic stem and progenitor cells (HSPCs) are derived from a subset of specialized endothelial cells, hemogenic endothelial cells, in the aorta-gonad-mesonephros region through endothelial-to-hematopoietic transition. HSPC generation is efficiently and accurately regulated by a variety of factors and signals; however, the precise control of these signals remains incompletely understood. Post-transcriptional regulation is crucial for gene expression, as the transcripts are usually bound by RNA-binding proteins (RBPs) to regulate RNA metabolism. Here, we report that the RBP protein Csde1-mediated translational control is essential for HSPC generation during zebrafish early development. Genetic mutants and morphants demonstrated that depletion of *csde1* impaired HSPC production in zebrafish embryos. Mechanistically, Csde1 regulates HSPC generation through modulating Wnt/β-catenin signaling activity. We demonstrate that Csde1 binds to *ctnnb1* mRNAs (encoding β-catenin, an effector of Wnt signaling) and regulates translation but not stability of *ctnnb1* mRNA, which further enhances β-catenin protein level and Wnt signal transduction activities. Together, we identify Csde1 as an important post-transcriptional regulator and provide new insights into how Wnt/β-catenin signaling is precisely regulated at the post-transcriptional level.

## Teaser

**Summary:** Loss of RNA-binding protein Csde1 leads to impaired development of hematopoietic stem and progenitor cells in zebrafish.

## INTRODUCTION

Hematopoietic stem and progenitor cells (HSPCs) are endowed with both self-renewal and multilineage differentiation properties (Laurenti and Gottgens, 2018; Orkin and Zon, 2008; Zhang et al., 2018). In vertebrates, the earliest HSPCs arise from the hemogenic endothelium within the ventral wall of the dorsal aorta (DA) in the aorta-gonad-mesonephros (AGM) region via the endothelial-to-hematopoietic transition (EHT) (Bertrand et al., 2010; Boisset et al., 2010; Kissa and Herbomel, 2010). This transition occurs within a narrow developmental window, during which endothelial and hematopoietic transcriptional programs are accurately coordinated to execute a shift in cell identity (Wu and Hirschi, 2021). Therefore, the precise orchestration of spatiotemporal gene expression is crucial to ensure the implementation of cell fate transition through EHT.

Several factors and signaling pathways have been identified as essential regulators for HSPC generation (Bigas et al., 2013; Orkin and Zon, 2008). Among these signals, Wnt/β-catenin signaling is an evolutionarily conserved signaling pathway that is involved in the regulation of embryonic HSPC development (Bigas et al., 2013; Gertow et al., 2013; Grainger et al., 2019, 2016; Richter et al., 2017; Woll et al., 2008). In the Wnt/β-catenin signaling (referred to as the canonical Wnt pathway) transduction process, Wnt ligands bind to Frizzled receptors, leading to β-catenin release from constitutive degradation, therefore promoting its stabilization and accumulation in cytoplasm. Then, β-catenin could be translocated into the nucleus and interact with LEF/TCF transcription factors to activate the transcription of target genes (Angers and Moon, 2009; MacDonald et al., 2009). Previous studies have demonstrated that β-catenin is expressed in most endothelial cells in the mouse dorsal aorta, but activated only in a subpopulation of aortic endothelium localized at the base of intra-aortic hematopoietic clusters (Ruiz-Herguido et al., 2012). Deletion of β-catenin from endothelial cells leads to decreased HSPC generation, whereas the activation of β-catenin increases HSPC production in explants of mouse AGM (Ruiz-Herguido et al., 2012) and zebrafish (Grainger et al., 2016). However, how Wnt/β-catenin signaling is spatial-temporally regulated in HSPC development remains elusive.

Accumulating evidence suggests that post-transcriptional regulation plays a fundamental role in controlling precise and rapid gene expression and therefore has an impact on hematopoiesis (de Rooij et al., 2019; Yuan and Muljo, 2013). Post-transcriptional regulatory processes are mainly mediated by RNA-binding proteins (RBPs), which modulate mRNA splicing, polyadenylation, localization, degradation and translation (Gehring et al., 2017; Moore, 2005). Cold shock domain containing E1 [Csde1, also known as Up-stream of N-Ras (UNR)] is a conserved cytoplasmic RBP with high affinity for purine-rich mRNAs (Guo et al., 2020; Ray et al., 2015; Triqueneaux et al., 1999). Csde1 plays a dual role in regulating the stability and translation of mRNAs (Chang et al., 2004; Cornelis et al., 2005; Dormoy-Raclet et al., 2005; Duncan et al., 2009; Mitchell et al., 2003; Ray and Anderson, 2016; Saltel et al., 2017; Schepens et al., 2007). Csde1 protein is involved in the regulation of diverse biological processes, including epithelial-to-mesenchymal transition (EMT) (Wurth et al., 2016), erythropoiesis (Horos et al., 2012; Moore et al., 2018a,b), tumorigenesis (Avolio et al., 2022; Wurth et al., 2016), neurogenesis (Ju Lee et al., 2017) and synapse development and transmission (Guo et al., 2019). However, it remains unknown whether Csde1-mediated post-transcriptional regulation plays a role in regulating developmental signals during embryonic HSPC development.

In the present study, we demonstrate that Csde1-mediated translational control is important for HSPC development in zebrafish. Loss of *csde1* leads to impaired HSPC generation. Further mechanistic studies demonstrate that Csde1 interacts with *ctnnb1* mRNA and promotes its translation, thus activating Wnt signal transduction in endothelial cells (ECs) during embryonic HSPC generation. Our study uncovers a pivotal role of post-transcriptional regulation in HSPC development.

## RESULTS

### Post-transcriptional regulatory processes are enriched in HECs and nascent HSPCs

To determine whether post-transcriptional regulation is involved in HSPC generation, we first evaluated module scores to measure the transcriptional levels of genes related to post-transcriptional regulatory processes to characterize the molecular features using published single-cell transcriptome data from endothelial and hematopoietic cells in zebrafish embryos at 36 h post fertilization (hpf), the timing of HSPC generation (Xia et al., 2023). The results revealed significantly increased scores for genes involved in post-transcriptional regulation of gene expression in hemogenic endothelial cells (HECs) and nascent HSPCs, compared with those in arterial endothelial cells (AECs). Moreover, genes involved in most post-transcriptional regulatory processes, including RNA stabilization, RNA modification, RNA splicing, poly (A) binding and translational initiation, also showed significant module scores in HECs and nascent HSPCs (Fig. S1A), suggesting their possible roles in HSPC generation. Furthermore, to elucidate the conserved role of post-transcriptional regulation, we re-analyzed publicly available human single-cell transcriptome data (Calvanese et al., 2022). Our analysis revealed that the biological processes associated with post-transcriptional regulation that were enriched in zebrafish HECs and HSPCs, also displayed high scores in human HECs and HSPCs (Fig. S1B). Together, these results showed that genes related to post-transcriptional regulatory processes are highly enriched in HECs and nascent HSPCs, suggesting their potential involvement in HSPC generation.

### Csde1 is required for HSPC generation during early development

Translation initiation and RNA stabilization are important in regulating gene expression during developmental processes (Buccitelli and Selbach, 2020; Kong and Lasko, 2012). Csde1 is previously reported as a regulator of mRNA stability and translation control (Ray et al., 2015), but little is known about its function in embryonic HSPC generation. Based on the results of whole mount *in situ* hybridization (WISH) and quantitative RT-PCR (qPCR), we found that *csde1* initially displayed maternal expression and later on was highly expressed in the AGM region from 24 to 36 hpf (Fig. S2A,B). Moreover, double fluorescence *in situ* hybridization (dFISH) and qPCR with sorted *fli1a*:EGFP^+^ ECs confirmed that *csde1* was expressed in ECs and *cmyb*^+^ (*myb*^+^) HSPCs in zebrafish embryos (Fig. S2C,D). Together, these results showed that the expression of *csde1* is enriched in the definitive hematopoiesis tissue during HSPC generation in zebrafish, indicating its potential role in developmental hematopoiesis.

To further investigate whether *csde1* is required for HSPC development, we first used CRISPR/Cas9 technology to generate a *csde1* mutant, in which a 1-bp deletion and 11-bp insertion in the fifth exon were identified, leading to a premature stop codon (Fig. S3A,B). Zygotic mutants were obtained by cross-mating *csde1*^+/−^ adult zebrafish, but only 2% of mutants survived to adulthood and they failed to produce maternal-zygotic mutant embryos. For this reason, we used *csde1* zygotic mutants to perform further investigation. WISH and western blotting revealed markedly reduced transcript and protein levels of Csde1, respectively, in the zygotic mutants (Fig. S3C,D). By contrast, no general developmental defects in neurogenesis (labeled by *elavl3*), somite development (labeled by *myod1*) and endothelial cells (labeled by *kdrl*) were observed in *csde1* zygotic mutants (Fig. S3E).

**Fig. 1.**
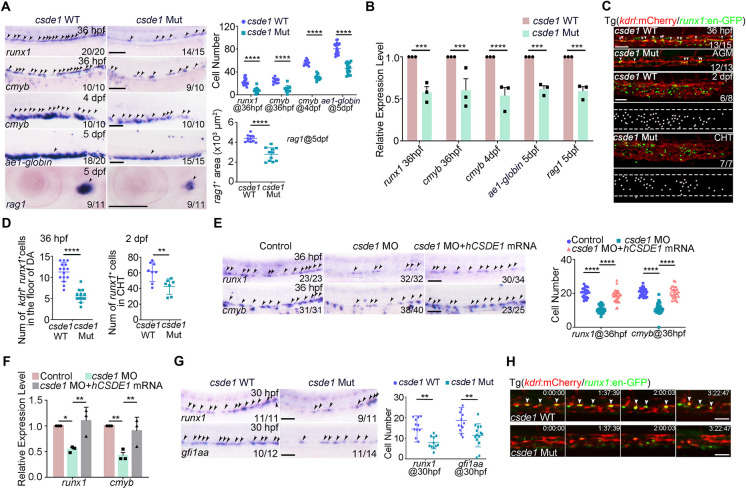
**HSPC generation is impaired in *csde1* mutants.** (A) Expression of HSPC markers *runx1* and *cmyb* (arrowheads) in the AGM region at 36 hpf, *cmyb* (arrowheads) in the CHT region at 4 dpf, erythroid marker *ae1-globin* and lymphoid marker *rag1* (arrowheads) in the thymus region at 5 dpf in *csde1* mutants and WT by WISH, with quantification (right panels). (B) qPCR analysis of *runx1*, *cmyb*, *ae1-globin* and *rag1* in *csde1* mutants and WT at 36 hpf, 4 dpf or 5 dpf. (C,D) Confocal imaging showing *kdrl*^+^ *runx1*^+^ HECs (white arrowheads) in the AGM region at 36 hpf and *runx1*^+^ HSPCs in the CHT region at 2 dpf in *csde1* mutants and WT (C) and quantification (D). Dashed lines in C outline the CHT region. (E,F) WISH (E, with quantification, right panel) and qPCR (F) analysis showing that the expression of *runx1* and *cmyb* (arrowheads) at 36 hpf was rescued by *hCSDE1* mRNA, compared with *csde1* morphants. (G) Examination of the HEC marker *runx1* and *gfi1aa* expression in WT and *csde1* mutants at 30 hpf by WISH, with quantification (right panel). (H) Snapshot of EHT (arrowheads) in *csde1* mutants and siblings. Data are mean±s.d. *P<0.05, **P<0.01, ***P<0.001, ****P<0.0001 (two-tailed unpaired Student's *t*-test). *n*≥3 replicates. Numbers indicate the number of embryos with respective phenotype/total number of embryos analyzed in each experiment (A,C,E,G). Scale bars: 100 μm (A,E,G); 50 μm (C,H).

Next, we examined embryonic hematopoiesis and WISH revealed that pre-hematopoietic mesoderm at the 5- and 10-somite stage [labeled by *fli1a* (*fli1*), *scl* (*tal1*), *lmo2* and *kdrl*] (Fig. S4A), primitive hematopoiesis at 24 hpf [labeled by *gata1a* for primitive erythroid and *pu.1* (*spi1b*) for primitive myeloid] (Fig. S4B) and erythroid-myeloid precursor (EMP)-derived erythrocytes [labeled by *gata1a* and *ae1-globin* (*hbae1.1*)] and myeloid cells (labeled by *mpx* and *mpeg1.1*) at 36 and 48 hpf were unaffected upon *csde1*-deficiency (Fig. S4C-F). By contrast, at 36 hpf, HSPC production (labeled by *runx1* and *cmyb*) was significantly decreased when measured using WISH and qPCR (Fig. 1A,B). Consequently, *csde1*-deletion-induced HSPC defect further led to a reduced number of HSPCs in the caudal hematopoietic tissue (CHT) region [labeled by *cmyb* at 4 days postfertilization (dpf)] and impaired differentiation towards lymphoid (labeled by *rag1* in the thymus at 5 dpf) and erythroid (labeled by *ae1-globin* at 5 dpf) cells (Fig. 1A,B). Furthermore, live imaging revealed that the number of precursors (*kdrl*^+^*runx1*^+^) at 36 hpf and *runx1*^+^ HSPCs at 2 dpf in *csde1* zygotic mutants in the Tg(*kdrl*:mCherry;*runx1*:en-GFP) background was significantly lower than that of wild-type (WT) (Fig. 1C,D). Thus, these data supported Csde1 being required for HSPC development in zebrafish.

To confirm the aforementioned genetic mutant phenotypes, we used *csde1* ATG morpholino (MO) anti-sense oligomers to knock down endogenous *csde1* expression and validated the efficiency using western blotting (Fig. S5A). Similar to *csde1* mutants, WISH showed that HSPC production was impaired in *csde1* morphants (Fig. S5B-D). Importantly, overexpression of *csde1* mRNA (without *csde1* MO binding site) or human *CSDE1* mRNA, both escaping from *csde1* MO blocking, could efficiently rescue the impaired HSPC development (Fig. S5B,C; Fig. 1E,F), indicating that loss of *csde1* is responsible for the observed HSPC phenotypes and also supporting that *csde1* may play a conserved role in both zebrafish and humans.

Because the earliest HSPCs are derived from HECs, the observed HSPC defects in *csde1*-deficient embryos were likely attributed to the impaired HECs. To explore this possibility, we examined HEC markers *runx1* and *gfi1aa*. WISH showed that the expression of *runx1* at 24 and 26 hpf was only slightly reduced in *csde1* mutants (Fig. S5E), however, at 30 hpf the expression of *runx1* and *gfi1aa* was evidently compromised (Fig. 1G), suggesting that HEC specification was disrupted in the absence of *csde1*. Furthermore, lineage tracing of EHT by timelapse imaging showed considerably fewer EHT events in *csde1* mutants than seen in WT embryos (Fig. 1H; Movies 1 and 2), suggesting that Csde1 is required for HEC specification and emergence of HSPCs. Taken together, our results demonstrated that the impaired HSPC development in *csde1*-deficient embryos is attributed to defects in HECs.

### Csde1 regulates HSPC specification in an EC-autonomous manner

**Fig. 2.**
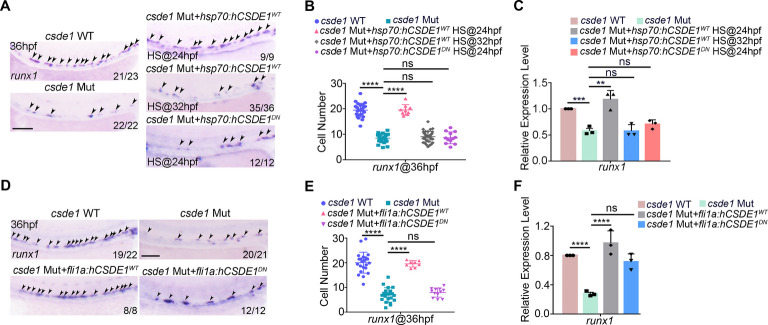
**Csde1 regulates HSPC specification in an EC autonomous manner.** (A) WISH analysis showing the expression of *runx1* (arrowheads) in WT, *csde1* mutants and *csde1* mutants injected with *hsp70*: *flag*-*hCSDE1^WT^-*EGFP or *hsp70*: *flag*-*hCSDE1^DN^-*EGFP constructs at 36 hpf. (B) Quantification of the WISH data in A. (C) qPCR analysis of *runx1* in WT, *csde1* mutants and *csde1* mutants injected with *hsp70*: *flag*-*hCSDE1^WT^-*EGFP or *hsp70*: *flag*-*hCSDE1^DN^-*EGFP constructs at 36 hpf. (D) WISH analysis showing the expression of *runx1* (arrowheads) in WT, *csde1* mutants and *csde1* mutants injected with *fli1a*: *flag*-*hCSDE1^WT^-*EGFP or *fli1a*: *flag*-*hCSDE1^DN^-*EGFP constructs at 36 hpf. (E) Quantification of the WISH data in D. (F) qPCR analysis of *runx1* in WT, *csde1* mutants and *csde1* mutants injected with *fli1a*: *flag*-*hCSDE1^WT^-*EGFP or *fli1a*: *flag*-*hCSDE1^DN^-*EGFP constructs at 36 hpf. Data are mean±s.d. **P<0.01, ***P<0.001, ****P<0.0001 (two-tailed unpaired Student's *t*-test). ns, not significant. *n*≥3 replicates. Numbers indicate the number of embryos with respective phenotype/total number of embryos analyzed in each experiment (A,D). Scale bars: 100 μm.

To further determine when and how HSPC defects occurred in *csde1*-deficient embryos, we first applied the heat-shock (HS) inducible *hCSDE1^WT^*-EGFP overexpression system and detected strong EGFP expression at 36 hpf after HS (Fig. S5F). Western blotting revealed an efficient rescue effect on the decrease of Csde1 level in *csde1* morphants (Fig. S5G). WISH and qPCR showed that overexpression of *hCSDE1^WT^* at 24 hpf, but not 32 hpf, could efficiently restore the impaired HSPC generation in *csde1* mutants (Fig. 2A-C; Fig. S5H-J), suggesting that Csde1 functions in the critical phase of HSPC specification. Next, to investigate whether Csde1 is EC-autonomously required for HSPC development, we generated a construct driven by *fli1a* promoter to express EGFP-tagged *hCSDE1^WT^* (Fig. S5F). WISH and qPCR results showed that endothelial overexpression of *hCSDE1^WT^* had a significant rescue effect on HSPC defects in *csde1* mutants (Fig. 2D-F; Fig. S5K-M), indicating its EC-specific role.

Considering that Csde1 is an RNA-binding protein (Triqueneaux et al., 1999; Wurth et al., 2016), we further examined whether it is dependent upon the RNA-binding activity. To address this question, we generated dominant-negative (DN) human CSDE1 (*hCSDE1^DN^*) with an amino acid mutation in the conserved cold shock domain (Fig. S5N), which affected its mRNA binding affinity (Ray and Anderson, 2016). The *in vitro* streptavidin-biotin pull-down assay showed that the level of Flag-hCSDE1^DN^ pulled down by the biotin-probe was obviously decreased, compared with Flag-hCSDE1^WT^ (Fig. S5O), confirming the weak RNA binding activity of hCSDE1^DN^. Rescue experiments showed that neither HS-induced overexpression at 24 hpf, nor EC-specific expression of hCSDE1^DN^ could restore the impaired HSPC generation (Fig. 2A-F; Fig. S5H-M), suggesting that the regulatory role of Csde1 in HSPC generation is dependent upon its RNA-binding activity.

### β-Catenin is a direct target of Csde1

**Fig. 3.**
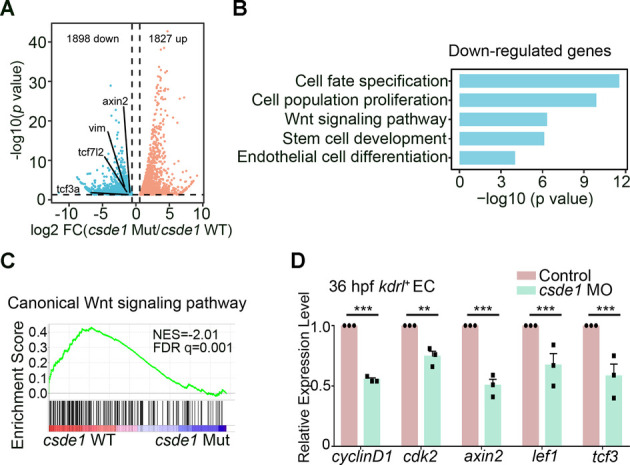
**Wnt signaling is downregulated upon *csde1* deficiency.** (A) Volcano plots showing the differentially expressed genes in ECs between WT siblings and *csde1* mutants. (B) Gene ontology analysis for the downregulated genes in *csde1* mutants, compared with WT siblings. (C) GSEA analysis of genes associated with canonical Wnt signaling pathway in *csde1* mutants compared with WT siblings. (D) qPCR analysis of Wnt signaling genes *cyclin D1*, *cdk2*, *axin2*, *lef1* and *tcf3* in ECs in control and *csde1* morphants at 36 hpf. Data are mean±s.d. **P<0.01, ***P<0.001 (two-tailed unpaired Student's *t*-test). *n*=3 replicates.

To investigate the underlying molecular mechanisms of Csde1 activity during HSPC development, RNA-seq was performed on *kdrl*^+^ ECs from *csde1* mutants and WT embryos at 33 hpf. According to the bioinformatics analysis, 1898 and 1827 genes were down- and upregulated, respectively, in *csde1* mutants, compared with WT controls (Fig. 3A). Gene ontology (GO) analysis revealed that genes with downregulated expression were enriched in various developmental processes and signals, including cell fate determination, cell proliferation, Wnt signaling pathway, stem cell development and endothelial cell differentiation (Fig. 3B). Among these signals, Wnt signaling has been demonstrated to be crucial in regulating embryonic HSPC generation in vertebrates (Goessling et al., 2009; Richter et al., 2017; Ruiz-Herguido et al., 2012; Sturgeon et al., 2014). Therefore, we speculated that Wnt signaling might be involved in *csde1* deficiency-induced HSPC defects. To explore this possibility, we performed gene set enrichment analysis (GSEA) and volcano plot analysis to further examine Wnt signaling. The results indicated that the canonical Wnt signaling pathway was significantly impaired (Fig. 3C) and Wnt-related genes, including *axin2*, *tcf7l2* and *tcf3a*, were downregulated (Fig. 3A) in *csde1* mutants. Furthermore, qPCR analysis confirmed the decreased expression of Wnt target genes in *csde1* morphants (Fig. 3D), supporting the impaired Wnt activity upon *csde1* deficiency. Thus, these findings indicated that loss of Csde1 attenuates Wnt signaling during HSPC generation.

**Fig. 4.**
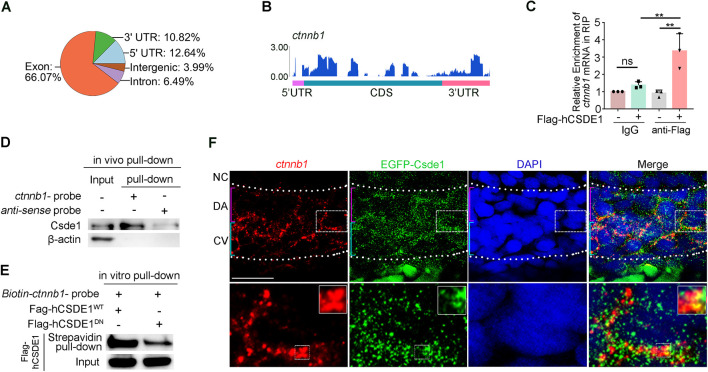
**Csde1 interacts with *ctnnb1* mRNA in ECs.** (A) Pie chart depicting the distribution of Csde1 binding peaks. (B) RIP-seq reads distribution of *ctnnb1* mRNA compared with control. Blue peaks indicate Csde1 binding sites. (C) RIP-qPCR analysis showing relative mRNA level of *ctnnb1* in IgG and anti-Flag groups. Data are mean±s.d. **P<0.01 (two-tailed unpaired Student's *t*-test). ns, not significant. *n*=3 replicates. (D) Western blotting showing that endogenous Csde1 protein can be efficiently pulled down by *ctnnb1* mRNA compared with anti-sense mRNA probe. (E) Western blotting showing the Flag-hCSDE1 protein from *Flag-hCSDE1^WT^*- or *Flag-hCSDE1^DN^*-transfected HEK293 cells pulled down with biotin-labeled *ctnnb1* probe. (F) The endogenous *ctnnb1* mRNA was detected by FISH, and overexpressed EGFP-Csde1 was detected by IF using an EGFP antibody in zebrafish at 36 hpf. The white dotted lines mark DA and CV regions. The bottom panels are magnifications of the dashed boxed areas (upper panels) showing the ECs with colocalization of EGFP-Csde1 and *ctnnb1* mRNA (white squares). DA, dorsal aorta; CV, cardinal vein; NC, notochord. Scale bars: 15 μm.

To further verify the role of Csde1 in the regulation of Wnt signaling, we performed RNA immunoprecipitation (RIP)-seq to detect direct targets of Csde1 (Fig. S6A). Similar to the distribution pattern within target mRNAs observed in previous mammalian studies (Avolio et al., 2022; Wurth et al., 2016), the majority of Csde1 binding peaks occurred within the coding sequence (CDS), 3′ untranslated region (UTR), and 5′ UTR (Fig. 4A). In total, 2756 Csde1 targets were identified, 519 of which were shown to be differentially expressed upon *csde1* deficiency (Fig. S6B). These transcripts are normally enriched during processes such as axonogenesis and morphogenesis (Fig. S6C). Importantly, the majority of targets (2237/2756) remained unaffected at the mRNA level upon *csde1*-depletion (Fig. S6B). As Csde1 has been shown to regulate translation initiation (Ray and Anderson, 2016), we speculated that these targets may be regulated at the translation level. Furthermore, GO analysis of these targets revealed enrichment in categories related to translation, mRNA metabolic process, mitotic cell cycle process and others (Fig. S6C), suggesting the conserved functions of Csde1 in mammals (Wurth et al., 2016).

Importantly, among these targets, *ctnnb1* mRNA (encoding β-catenin) was identified with Csde1 binding peaks (Fig. 4B), suggesting that *ctnnb1* acts as a potential downstream target of Csde1. To further determine whether Csde1 could bind to *ctnnb1* mRNA, we performed RIP-qPCR and RNA pull-down assays. Flag-hCSDE1 RIP-qPCR analysis showed that *ctnnb1* mRNA was significantly enriched by CSDE1 (Fig. 4C). The *in vivo* pull-down assay showed that Csde1 protein can be efficiently pulled down by *ctnnb1* mRNA compared with control mRNA (Fig. 4D), suggesting the direct interaction between Csde1 and *ctnnb1* mRNA. Moreover, *in vitro* pull-down assay using biotin-labeled *ctnnb1* probes showed that Flag-hCSDE1^WT^ could be markedly pulled down by probes, compared with Flag-hCSDE1^DN^ (Fig. 4E), further confirming that Csde1 could bind to *ctnnb1* mRNA. To further observe the localization of Csde1 and *ctnnb1* mRNA in zebrafish embryos, we expressed EGFP-Csde1 and detected its expression by immunofluorescence (IF), and used FISH to detect endogenous *ctnnb1* mRNA. The results showed that EGFP-Csde1 colocalized with *ctnnb1* mRNA in the cytoplasm of ECs in the AGM region of 36 hpf embryos (Fig. 4F). Taken together, these results suggested that *ctnnb1* is a direct target of Csde1 in ECs in zebrafish embryos.

### Csde1 regulates Wnt signaling activity via translational control of β-catenin

**Fig. 5.**
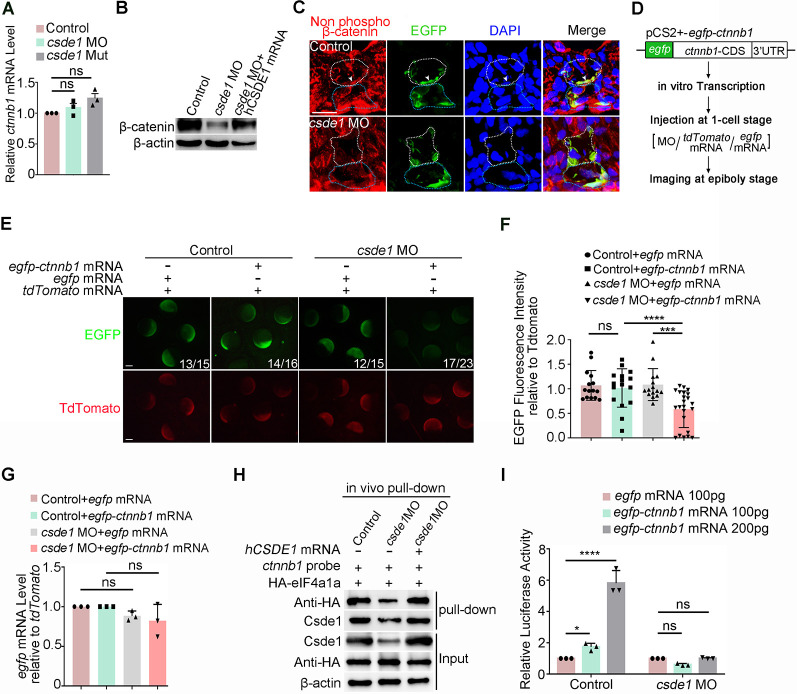
**Csde1 is involved in the translational regulation of β-catenin.** (A) qPCR analysis of *ctnnb1* expression in ECs in control, *csde1* morphants and *csde1* mutants at 36 hpf. (B) The protein level of β-catenin in control, *csde1* morphants and embryos co-injected with *csde1 atg*MO and *hCSDE1* mRNA at 28 hpf. (C) Immunofluorescence showing that nonphosphorylated β-catenin expression in ECs was reduced in *csde1* morphants. The white and blue dotted lines mark dorsal aorta and cardinal vein, respectively. The arrowhead denotes nuclear staining in the ECs located in the ventral wall of dorsal aorta. (D) Illustration of the EGFP-*ctnnb1* reporter and experimental procedure for reporter assay. (E,F) Representative images (E) and quantification (F) of the relative expression levels of EGFP and EGFP-*ctnnb1* with or without *csde1* deficiency at the 75% epiboly stage of development. *tdTomato* mRNA was co-injected into embryos with *egfp* mRNA or *egfp-ctnnb1* mRNA as injection control. (G) qPCR analysis showing the expression of *egfp* in control embryos and *csde1* morphants at the 75% epiboly stage. (H) *In vivo* transcribed *ctnnb1* mRNA pull-down assays followed by immunoblot analysis of anti-Csde1, anti-HA and anti-β-actin in control- and *csde1* MO-injected embryos. (I) TOPFlash luciferase reporter assays in *ctnnb1* mRNA-injected embryos with or without *csde1 atg*MO injection. Data are mean±s.d. *P<0.05, ***P<0.001, ****P<0.0001 (two-tailed unpaired Student's *t*-test). ns, not significant. *n*≥3 replicates. Numbers indicate the number of embryos with respective phenotype/total number of embryos analyzed in each experiment (E). Scale bars: 15 μm (C); 150 μm (E).

We next asked how Csde1 regulates the expression of β-catenin. Given that Csde1 functions as a regulator of mRNA stability and translation initiation (Ray et al., 2015), we first examined the transcript and protein levels of β-catenin in *csde1*-deficient embryos. qPCR in sorted ECs revealed comparable *ctnnb1* expression levels in control and *csde1*-deficient embryos (Fig. 5A). Conversely, the protein level of β-catenin was markedly decreased upon *csde1* depletion and could be rescued by overexpression of *hCSDE1* mRNA (Fig. 5B). IF using Tg (*fli1a*: EGFP) showed that the level of nonphosphorylated β-catenin in ECs was evidently reduced in *csde1* morphants (Fig. 5C). Besides, the *ctnnb1* mRNA levels remained unaltered in *csde1*-deficient embryos after α-amanitin treatment (RNA polymerase II inhibitor), compared with that in controls (Fig. S7A), suggesting that Csde1 is not required for *ctnnb1* mRNA stability, but likely acted at the translational level.

To address whether Csde1 is involved in the translational regulation of β-catenin, we performed a reporter assay in which the CDS and 3′ UTR region of *ctnnb1* containing Csde1-binding sites was fused with an EGFP tag (Fig. 5D). The *in vitro* transcribed reporter mRNAs were injected into control embryos or *csde1* morphants at the one-cell stage and the fluorescence intensity was monitored at the 75% epiboly stage (Fig. 5D). The results showed that the EGFP density in *csde1* morphants was significantly lower than that observed in controls (Fig. 5E,F), whereas the expression level of *egfp* mRNA was not affected by the status of *csde1* (Fig. 5G). These results indicate that the translation of β-catenin was likely regulated by Csde1. Furthermore, to determine how Csde1 affects β-catenin translation, we analyzed the binding of eukaryotic initiation factors (eIFs), which are essential for translation initiation (Sonenberg and Hinnebusch, 2009), in *ctnnb1* mRNA using pull down assay. Our results revealed that eIF4a1a protein (the homologue of eIF4a in mammals, the enzymatic core of the eIFs; Sonenberg and Hinnebusch, 2009) pulled down by *ctnnb1* was markedly decreased in the absence of *csde1*, compared with that in WT embryos (Fig. 5H). Moreover, the attenuated interaction of *ctnnb1* with eIF4a1a protein upon *csde1*-deficiency could be efficiently restored by overexpression of *hCSDE1* mRNA (Fig. 5H). These results suggested that Csde1 affects the interaction between translation initiation factors and *ctnnb1* mRNA, which then regulates β-catenin translation.

Furthermore, we examined Wnt signaling activity using TOPFlash reporter assays in zebrafish embryos. Briefly, *in vitro*-transcribed *ctnnb1* mRNA or *wnt3a* mRNA and TOPFlash constructs were co-injected into control embryos or *csde1* morphants at the one-cell stage, and the luciferase activity was monitored at the shield stage. Our results showed that Wnt signaling was dose-dependently induced by *ctnnb1* or *wnt3a* in control embryos and, conversely, knockdown of *csde1* inhibited Wnt activity (Fig. 5I; Fig. S7B), suggesting that Csde1 positively regulates Wnt/β-catenin signaling. Taken together, we concluded that Csde1 regulates Wnt signaling activity via translational control of β-catenin.

### Csde1 regulates HSPC generation through Wnt/β-catenin signaling

**Fig. 6.**
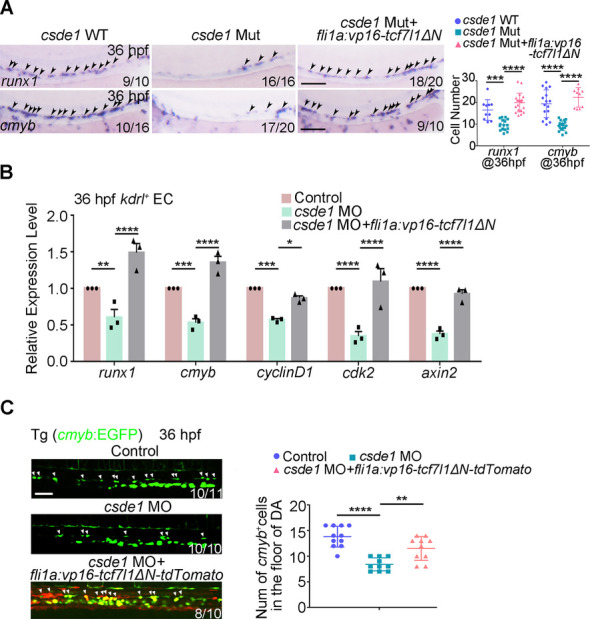
**Csde1 regulates Wnt/β-catenin signaling activity to control HSPC generation.** (A) Expression of *runx1* and *cmyb* (arrowheads) in the AGM region in WT siblings, *csde1* mutants and *csde1* mutants injected with *fli1a*:*vp16-tcf7l1ΔN*-tdTomato constructs at 36 hpf by WISH (left panels) with quantification (right panel). (B) qPCR showing the expression of *runx1*, *cmyb* and Wnt signaling genes at 36 hpf in control embryos, *csde1* morphants and *csde1* morphants injected with *fli1a*:*vp16-tcf7l1ΔN*-tdTomato constructs at 36 hpf. (C) Confocal imaging (left panels) showing that endothelial-derived*-tcf7l1ΔN-*tdTomato overexpression rescued the population of *cmyb*^+^ HSPCs (white arrowheads), compared with *csde1* morphants at 36 hpf, with quantification (right panel). Data are mean±s.d. *P<0.05, **P<0.01, ***P<0.001, ****P<0.0001 (two-tailed unpaired Student's *t*-test). *n*≥3 replicates. Numbers indicate the number of embryos with respective phenotype/total number of embryos analyzed in each experiment (A,C). Scale bars: 100 μm (A); 50 μm (C).

To determine whether the reduced Wnt activity accounts for HSPC defects in *csde1*-deficient embryos, we performed rescue experiments to enhance Wnt activity using constitutively activated TCF (VP16-Tcf7l1ΔN, a β-catenin-independent fusion protein without a β-catenin-binding site) (MacDonald et al., 2009; Zhang et al., 2020). Firstly, we demonstrated that EC-specific expression of *vp16-tcf7l1ΔN* efficiently induced the expression of Wnt target genes (Fig. S7C). Importantly, expression of *vp16-tcf7l1ΔN* in ECs was sufficient to rescue the decreased HSPCs (labeled by *runx1* and *cmyb*) in *csde1* mutants (Fig. 6A,B) and morphants (Fig. S7D), as well as the decreased expression of Wnt targets, including *cyclin D1*, *cdk2* and *axin2* (Fig. 6B). Furthermore, live imaging and quantitative analysis showed that the EC-specific *vp16-tcf7l1ΔN*-tdTomato overexpression restored the *cmyb*-EGFP^+^ HSPC population in the AGM region in *csde1* morphants (Fig. 6C), indicating that impaired endothelial Wnt signaling activity was responsible for HSPC defects in the absence of *csde1*. Taken together, we conclude that Csde1 plays a crucial role in HSPC generation by regulating the Wnt/β-catenin signaling pathway.

## DISCUSSION

Generation of HSPCs from HECs, including activation of the hematopoietic program and downregulation of the endothelial program, requires global reprogramming of regulatory networks involved in gene expression (Clements and Traver, 2013; Ding et al., 2021a; Wu and Hirschi, 2021). As a crucial regulatory mechanism, post-transcriptional regulation is essential for precise temporal and spatial modulation of gene expression (Gehring et al., 2017; Moore, 2005). However, comprehensive analysis of post-transcriptional regulation in HSPC generation remains elusive. In the present study, we analyzed the transcriptomes of arterial ECs, HECs and nascent HSPCs during EHT in zebrafish and humans, and found that many post-transcriptional regulatory programs were enriched in HECs and nascent HSPCs, indicating the activation of post-transcriptional regulation during EHT.

**Fig. 7.**
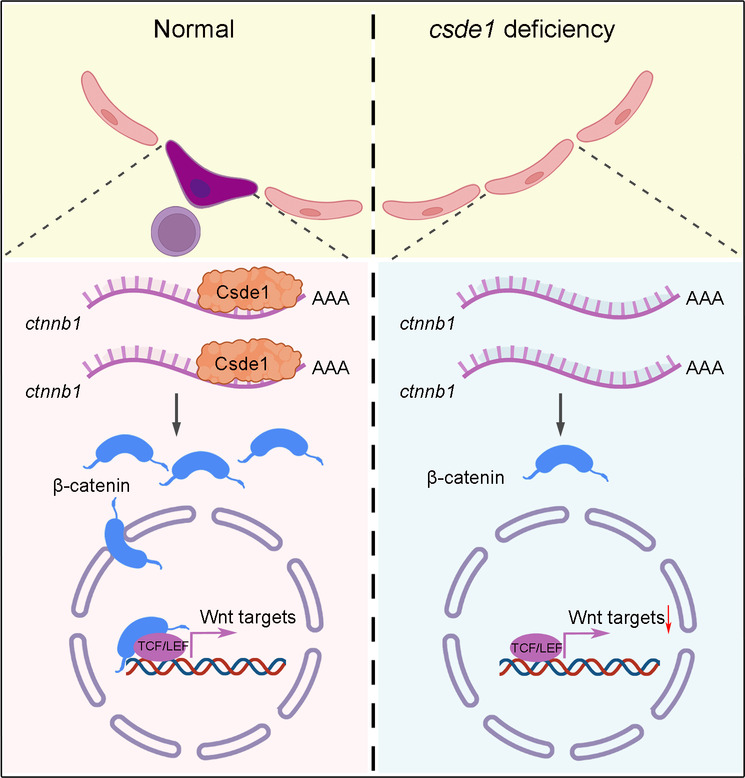
**Schematic showing that Csde1 modulates HSPC development via translational control of *ctnnb1* mRNA.** Csde1 binds to *ctnnb1* mRNA to regulate translation, further enhancing β-catenin protein level and Wnt signal transduction. In the absence of Csde1, the β-catenin protein level is reduced, which results in the downregulation of Wnt signaling, further leading to definitive hematopoiesis defects.

Recent studies demonstrated the important role of post-transcriptional regulation in embryonic hematopoiesis. For example, RNA m^6^A methyltransferase Mettl3, as a critical RBP, modulates HSPC specification by mediating RNA modification in zebrafish and mouse systems (Lv et al., 2018; Zhang et al., 2017). A very recent study verified the regulatory role of Cpeb1b-mediated cytoplasmic polyadenylation in embryonic HSPC development (Heng et al., 2023). Lin28b has been shown to exert wide-ranging effects on hematopoiesis, either via affecting *let-7* microRNA stability or by regulating mRNA translation (Basak et al., 2020; Wang et al., 2022). Here, we identified Csde1 to be a novel post-transcriptional regulator in controlling HSPC generation through modulating β-catenin translation and Wnt signaling activity in endothelial cells in zebrafish embryos (Fig. 7).

The role of Wnt/β-catenin signaling in embryonic HSPC generation has been the subject of many studies. Previously, a study using a zebrafish model verified that prostaglandin E2 positively regulates HSC formation in the AGM in a β-catenin-dependent manner, which demonstrated the regulatory role of β-catenin in HSPC generation (Goessling et al., 2009). In addition, in murine HSPC development, deletion of β-catenin in the embryonic endothelium precludes HSPC generation, whereas deletion in hematopoietic cells does not have any obvious effect. Inhibitor treatment demonstrated a time- and dose-dependent manner of β-catenin activity in HSPC production (Ruiz-Herguido et al., 2012). Activation of Wnt signaling by somite-derived Wnt9a promotes HSPC generation (Grainger et al., 2019, 2016). Altogether, these studies showed that Wnt/β-catenin signaling is required for the emergence of HSPCs. However, little is known about how Wnt signaling is activated sufficiently and precisely to complete cell fate transition in the EHT (Fig. 7). Here, we discovered that Csde1-mediated translational control in ECs is essential for ensuring sufficient β-catenin protein expression, which subsequently responds to cellular signals and activates targets in the EHT (Fig. 7). We showed enriched expression of *csde1* in ECs/HSPCs during HSPC generation and colocalization of Csde1 protein and *ctnnb1* mRNA in ECs, suggesting the timing and cell specificity. We further demonstrated that Csde1 interacts with translation initiation factors to promote β-catenin translation, suggesting the direct and specific regulation of β-catenin by Csde1. Our functional assays, together with the RIP-seq and RNA-seq analyses, also demonstrated that Csde1 regulates β-catenin at the translational level. Consistent with our findings here, previous studies using fibroblasts from human patients (El Khouri et al., 2021), mouse cortical neurons (Guo et al., 2019) and melanoma cells (Wurth et al., 2016) also showed that depletion of CSDE1 reduced β-catenin expression at the protein level.

In summary, we demonstrated that Csde1-mediated post-transcriptional regulation is crucial for HSPC generation in vertebrates. These findings deepen our understanding of HSPC specification, and provide new insight into improving existing methods for induction of functional HSPCs *in vitro*.

## MATERIALS AND METHODS

### Zebrafish strains and maintenance

Zebrafish strains including Tübingen, Tg(*kdrl*:mCherry) (Bertrand et al., 2010), Tg(*cmyb*:EGFP) (North et al., 2007), Tg(*runx1*:en-GFP) (Zhang et al., 2015), Tg(*fli1a*:EGFP) (Lawson and Weinstein, 2002), Tg(*kdrl*:EGFP) (Jin et al., 2007) and *csde1* mutants were raised in system water at 28.5°C. The embryos and larvae were obtained through natural spawning as previously described (Li et al., 2022). This study was approved by the Ethical Review Committee of the Institute of Hematology, Chinese Academy of Medical Sciences.

### Morpholino, mRNA and plasmid microinjection

The *csde1* ATG morpholino (*atg*MO) was purchased from Gene Tools. The sequence is 5′-GGGTCAAAACTCATCTTGTTCTGTT-3′. We injected 5 ng *atg*MOs into one-cell stage embryos. For *in vitro* transcription, zebrafish full-length mismatched *csde1*, full-length CDS of human *CSDE1* (*hCSDE1*), *egfp*, or zebrafish *ctnnb1* (including CDS and 3′ UTR) fused with *egfp* (*egfp-ctnnb1*) were cloned into pCS2^+^ vector. mRNAs were synthesized using the mMessage Machine SP6 transcription kit (AM1340, Ambion) and injected into one-cell stage zebrafish embryos. For overexpression experiments, *hCSDE1^WT^*, *hCSDE1^DN^* or *vp16-tcf7l1ΔN* were cloned into a pDestTol2pA2 vector with *fli1a* promoter or *hsp70* promoter and an EGFP/tdTomato reporter from DNA Assembly (E2621S, NEBuilder). These constructs (25-40 pg) with *tol2* mRNA (25-35 pg) were co-injected into the one-cell stage embryos as previously described (Kwan et al., 2007). The primers used for cloning are listed in Table S1.

### Generation of *csde1* mutant using CRISPR/Cas9

The guide RNA (gRNA) of *csde1* was designed in https://zlab.bio/guide-design-resources and synthesized by T7 RNA polymerase (P2075, Promega) using an *in vitro* transcription system as previously described (Xue et al., 2017). The sequence of *csde1* gRNA was 5′-GGGAGTGGTTTGTGCTACCAAGG-3′. A 518 bp DNA fragment spanning the gRNA target site was amplified and then sequenced to validate the mutations. The detailed *csde1* primers used for mutation validation are listed in Table S1.

### WISH and dFISH

WISH and dFISH were performed as previously described (Jowett and Yan, 1996; Liu and Patient, 2008; Zhang et al., 2017). In brief, the zebrafish embryos were fixed with 4% paraformaldehyde (PFA, P6148, Sigma-Aldrich) in PBS at 4°C overnight. A hybridization was performed using hybridization buffer containing digoxigenin (Dig)-labeled probes at 65°C overnight. After washing, the embryos were incubated with anti-Dig-AP antibody solution (11093274910, Roche, 1:5000) and stained with BM purple (11442074001, Roche). The dFISH was carried out with fluorescence (Flu)-labeled probes and Dig-labeled probes. The embryos were then incubated with anti-Flu-POD (11426346910, Roche, 1:100) or anti-Dig-POD (11633716001, Roche, 1:100) antibodies. The probes used in this study include *csde1*, *cmyb*, *runx1*, *gfi1aa*, *gata1a*, *rag1*, *pu.1*, *ae1-globin*, *lmo2*, *fli1a*, *scl*, *mpeg1.1*, *mpx*, *myod1*, *kdrl*, *elavl3* and *ctnnb1*.

### Western blotting

Western blotting was performed as previously reported (Zhang et al., 2017). In brief, the trunk regions of zebrafish embryos were homogenized in lysis buffer [10 mM Tris-HCl (pH 8.0), 10 mM NaCl, 0.5% NP-40] containing protease inhibitor (P8340, Sigma-Aldrich). The proteins were separated by 12% SDS-PAGE gel and then transferred to nitrocellulose membrane. After blocking with 5% bovine serum albumin (BSA), the samples were incubated with anti-Csde1 (HPA018846, Sigma-Aldrich, 1:1000), anti-β-catenin (8480, Cell Signaling Technology, 1:1000), anti-β-actin (4967, Cell Signaling Technology, 1:1000), anti-HA (3724T, Cell Signaling Technology, 1:1000) or anti-FLAG (F7425, Sigma-Aldrich, 1:1000) antibodies overnight at 4°C, respectively.

### Immunofluorescence

The control embryos and *csde1* morphants in Tg(*fli1a*:EGFP) or embryos injected with *egfp*-*Csde1* mRNA at 28 hpf were fixed with 4% PFA overnight at 4°C. After washing and permeabilizing, the whole embryos or transverse section were blocked with 1% BSA for 1 h and incubated with antibodies, including anti-GFP (66002-1-Ig, Proteintech, 1:500) and anti-β-catenin (8480, Cell Signaling Technology, 1:100) or anti-nonphosphorylated β-catenin (8814, Cell Signaling Technology, 1:200) at 4°C overnight. After washing, the embryos were incubated with Alexa Fluor™ 488 conjugated antibody (A11001, Thermo Fisher Scientific, 1:500) and Alexa Fluor™ 594 conjugated antibody (A11037, Thermo Fisher Scientific, 1:500) at 4°C overnight.

### RNA-seq and processing of data

To perform RNA-seq, *kdrl*^+^ EC cells were sorted from the trunk region of sibling WT embryos and *csde1* mutants with Tg(*kdrl*:EGFP) background at 33 hpf on a FACSAria™ III Cell Sorter (BD Biosciences). The total RNAs of *csde1* mutants and *csde1* WT were isolated using Trizol™ Reagent and the mRNA libraries were constructed as previously described (Ding et al., 2021b). The mRNA libraries were sequenced on an Illumina NovaSeq 6000 system with pair-end 150 bp (BerryGenomics). Sequenced reads were filtered to exclude adapters with Trim galore (version 0.6.7). The remaining sequences were aligned to the Zebrafish Genome (version GRCz11) using Hisat2 (version 2.2.1) with default parameters (Kim et al., 2019). Only uniquely mapped reads with mapping quality score ≥20 were kept using Samtools (version 1.9) for each sample. The number of aligned reads was counted using the HTSeq tool with parameters ‘--mode union --stranded no’ (version 0.13.5). Differential gene expression (DEG) between siblings and *csde1* mutants was analyzed using DESeq2 (version 1.28.1 under R version 4.0.5) with the method *P*-value <0.05 and absolute value of fold change >1.5. Enrichment of GO terms was performed using clusterProfiler (version 3.18.1) with default parameters and plotted in ggplot2 (version 3.3.3). GSEA (version 4.0.3) was performed as previously described (Subramanian et al., 2005). The annotated gene sets were selected from the Molecular Signatures Database (MSigDB version 7.5). The sequencing metrics for RNA-seq data are listed in Table S2.

### RNA immunoprecipitation

RIP was performed in control embryos and *hsp70:flag-hCSDE1-*EGFP embryos as described previously (Yang et al., 2019). The samples were incubated with Flag M2 magnetic beads (M8823, Sigma-Aldrich) or Protein A beads (10001D, Invitrogen) with mouse IgG at 4°C for 4 h. After washing eight times with 0.8 ml ice-cold NT2 buffer [200 mM NaCl, 50 mM HEPES (pH 7.6), 2 mM EDTA, 0.05% NP-40, 0.5 mM DTT and 0.4 U/µl RNase inhibitor], RNAs were fragmented into ∼200-300 nt by 2 U/ml Micrococcal Nuclease (MNase, M0247S, NEBuilder) in MN reaction buffer [50 mM Tris-HCl (pH 7.9) and 5 mM CaCl_2_] at 37°C, and then terminated with 1× PNK+EGTA buffer [50 mM Tris-HCl (pH 7.4), 20 mM EGTA, 0.5% NP-40 and 0.5 mM DTT]. After twice washing with ice-cold NT2 buffer and once with ice-cold 1× PK buffer [100 mM Tris-HCl (pH 7.4), 50 mM NaCl, 10 mM EDTA and 0.2% SDS], the proteins were digested with 4 μg/μl proteinase K (03115828001, Roche) for 1 h at 55°C. Finally, the RNAs were purified and precipitated by ethanol. The RNAs were then reverse transcribed and the amplified cDNAs were sequenced on Illumina NovaSeq 6000 system (Novogene) or used in RIP-qPCR.

### Processing of RIP-seq data

High-throughput sequencing data was filtered using Cutadapt (version 1.18) (Martin, 2011). Reads were aligned to the GRCz11 genome with Hisat2 (version 2.2.1) (Kim et al., 2015). After filtering low quality reads (q≥20) and merging replicates, MACS2 (version 2.2.7.1) was used for the peak calling with the options ‘--nomodel -g 1.4e9 --keep-dup all -B -p 1e-3’ (Zhang et al., 2008). Signal tracks were generated using bamCompare (version 3.5.1, deeptools) function and visualized by ggplot2 (Ramirez et al., 2014). According to the canonical University of California, Santa Cruz annotation of the zebrafish genome (Navarro Gonzalez et al., 2021), coordinates were assigned to genomic features.

### Module score analysis of single cell RNA-seq data

The module score, which could measure the average expression levels of a set of genes, was calculated using the Seurat function ‘AddModuleScore’ with the default parameters (Stuart et al., 2019). We used post-transcriptional regulation of gene expression, RNA stabilization, RNA modification, positive regulation of mRNA splicing via spliceosome, poly A binding and translational initiation gene sets from the Molecular Signatures Database (MSigDB, version 7.5).

### *In vivo* RNA pull-down assay

*In vivo* RNA pull-down assay was performed as in previous studies (Hovhannisyan and Carstens, 2007; Simon et al., 2011). Firstly, the *ctnnb1* RNA probe and antisense probe were synthesized using RNA polymerase at 37°C and purified with 6% denaturing urea polyacrylamide gel, then oxidized in a reaction mixture containing 0.1 M sodium acetate (pH 5.0) and 5 mM sodium periodate (311448, Sigma-Aldrich) for 1 h at room temperature. After precipitation by ethanol, the probes were resuspended in 500 µl 0.1 M sodium acetate (pH 5.0) and incubated with the sodium acetate-treated adipic acid dihydrazide agarose bead (A0802, Sigma-Aldrich) on a rotator at 4°C for 12 h. After washing, the RNA-bead complexes were incubated with the protein extracts at 30°C for 30 min. Finally, the complexes were washed and then analyzed by immunoblotting. The primers of the targets are shown in Table S1.

### *In vitro* RNA pull-down assay

The HEK293T cells were transfected with pcDNA3.1:*flag*-*hCSDE1^WT^* or pcDNA3.1:*flag*-*hCSDE1^DN^* using the PEI MAX (24765, Polysciences). After 48 h, the cells were lysed and sonicated for 15 min using a Sonic Dismembrator (Diagenode). After being heated at 65°C and flash-cooled on ice, the biotin-labeled RNA probes were incubated with cell exacts and pre-cleared streptavidin-conjugated magnetic beads (65001, Invitrogen) in binding buffer [50 mM Tris-HCl (pH 7.4), 150 mM NaCl, 0.5 mM EDTA, 0.1% NP-40, 1 mM DTT, protease inhibitor cocktail, 0.4 U/ml RNase inhibitor] for 1 h at 4°C. After washing, the bead-bound proteins were analyzed by immunoblotting. The RNA probes used for *in vitro* RNA pull-down assays were: Biotin probe, AAGAACAAGAAGAAGAACAA; Biotin-*ctnnb1* probe, AGAAGAAAGAAAGCCCCAAAAAAAAAAGAAG.

### Flow cytometry and fluorescence-activated cell sorting

The transgenic zebrafish embryos were collected and the trunk regions were dissected and digested into single cell suspension. The ECs (*kdrl*:mCherry^+^), HECs (*kdrl*:mCherry^+^;*runx1*:en-GFP^+^) and HSPCs (*runx1*:en-GFP^+^) were sorted using FACSAria™ III Cell Sorter (BD Biosciences).

### Reporter assays

For luciferase reporter assay, *egfp-ctnnb1* mRNA (0, 100, 200 pg), or 100 pg *egfp* mRNA (as a control), 100 pg TOPFlash constructs with 20 pg *Renilla* constructs were co-injected into one-cell stage embryos with or without 5 ng *csde1* MOs. The embryos were raised to the shield stage and lysed in 100 μl Passive Lysis Buffer. TOPFlash/*Renilla* luciferase assays were performed with the Dual-Luciferase Reporter Assay Kit (E1910, Promega) according to the manufacturer's instructions. The luciferase activity was measured by a microplate reader (Synergy HTX, BioTek).

For fluorescence reporter assay, 200 pg *egfp* mRNA or 200 pg *egfp-ctnnb1* mRNA was co-injected with *tdtomato* mRNA into the one-cell-stage embryos with or without *csde1* MOs. All plasmids were verified by sequencing.

### Chemical treatment

Control embryos and *csde1* morphants were treated with DMSO or 0.2 ng α-amanitin (HY19610, MedChem Express) at 24 hpf and then total RNAs were collected at 0, 2, 4 and 8 h after treatment. qPCR was performed as described above to validate the expression of *ctnnb1*.

### Confocal microscopy

The embryos were embedded in the 1% low-melting agarose on the dishes. Confocal images and EHT process were captured by Andor Dragonfly 505 confocal microscope (Oxford Instruments). The analysis of images was carried out by Imaris (Oxford Instruments) and ImageJ (National Institutes of Health).

### Statistical analysis

All statistical analyses of qPCR, confocal imaging, reporter assay and WISH results were performed on at least three independent biological or experimental replicates. The quantification of WISH data was carried out using ImageJ as described previously (Dobrzycki et al., 2020). The number of HSPCs in the CHT region was quantified using the software Bitplane Imaris 7.4.2 (Xue et al., 2017). The two-tailed unpaired Student's *t*-test was used for statistical analysis. All data are shown as mean±s.d. and analyzed using GraphPad PRISM 7 software. *P*-values were used for significance.

## Supplementary Material

10.1242/develop.201890_sup1 Supplementary information

Table S1. The primers for plasmid construction, genotyping and qPCR.

Table S2. The sequencing metrics for RNA-seq data.
