# Supplementary material for: The RNA-binding protein CSDE1 promotes hematopoietic stem and progenitor cell generation via translational control of Wnt signaling
Source: Development. 2023 Oct 24;150(21):dev201890. doi: 10.1242/dev.201890 (PMC10652045; doi:10.1242/dev.201890)
Supplement: Supplementary information [file develop-150-201890-s1.pdf]

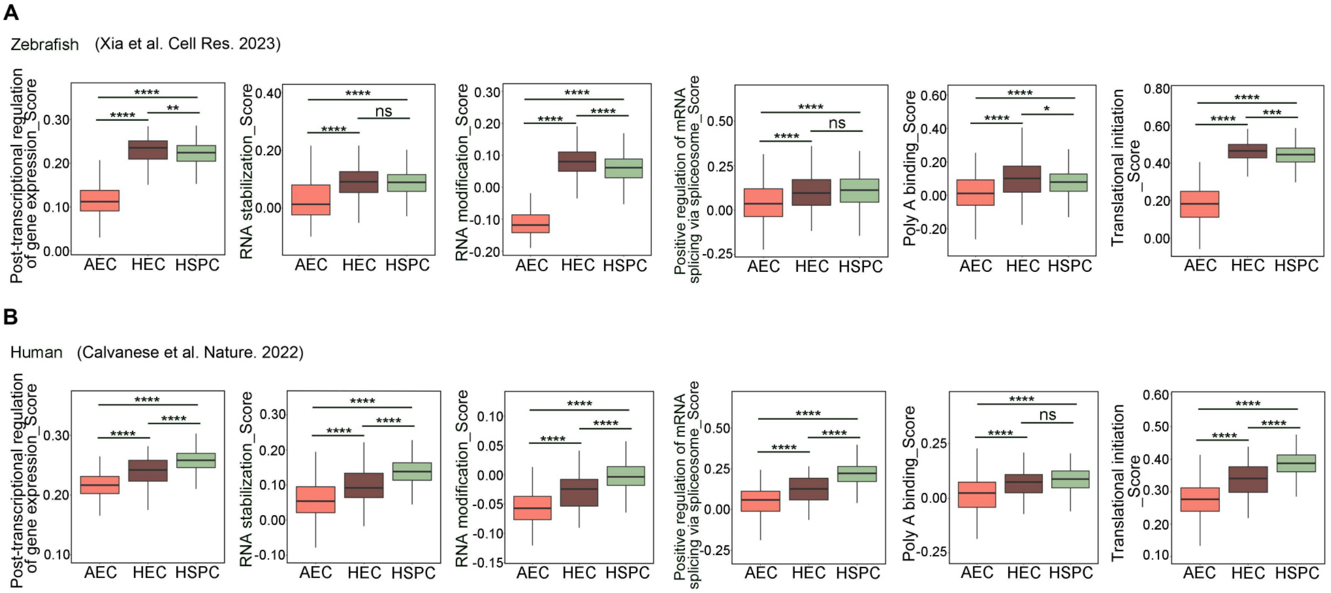

Fig. S1. **Post-transcriptional regulatory processes are enriched during EHT.** (A) (A) Module scores in genes involved in RNA stabilization, RNA modification, RNA splicing, poly A binding and translational initiation in zebrafish AECs, HECs and HSPCs.

(B) Module scores in genes involved in RNA stabilization, RNA modification, RNA splicing, poly A binding and translational initiation in human AECs, HECs and HSPCs. Error bars, mean ± s.d. *****p*<0.0001. NES, normalized enrichment score.

Development • Supplementary information

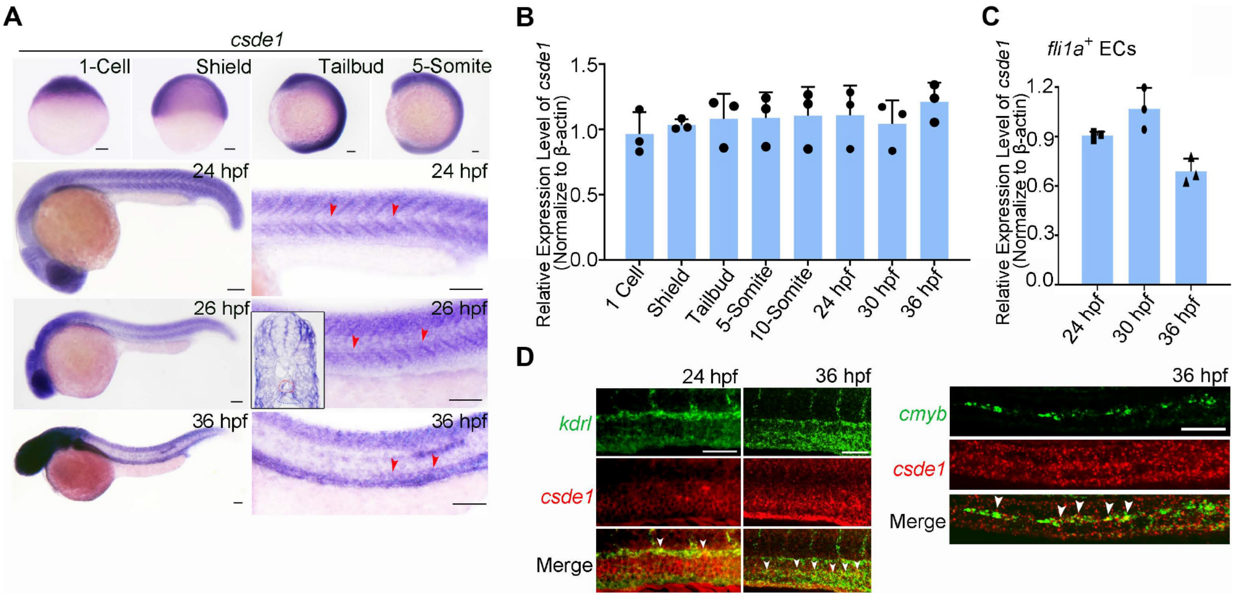

Fig. S2. Csde1 is expressed in ECs and HSPCs in AGM region.

(A) WISH showing the expression of *csde1* from 1-cell stage to 36 hpf. Red arrowheads marked the expression of *csde1* in AGM region at 24, 26, and 36 hpf. Transverse section post WISH at 26 hpf showed the expression of *csde1* in the endothelial cells. The red and blue dotted lines marked dorsal aorta and cardinal vein, respectively.

(B) qPCR showing the expression of *csde1* from 1-cell stage to 36 hpf.

(C) qPCR showing the expression of *csde1* in sorted *fli1a*:EGFP$^+$ ECs at 24, 30, and 36 hpf.

(D) Double FISH analysis showed that *csde1* was co-expressed with *kdrl* (white arrowheads) and *cmyb* (white arrowheads) in the AGM region at 24 and 36 hpf. Scale bars, 50 µm. n=3 replicates.

Development • Supplementary information

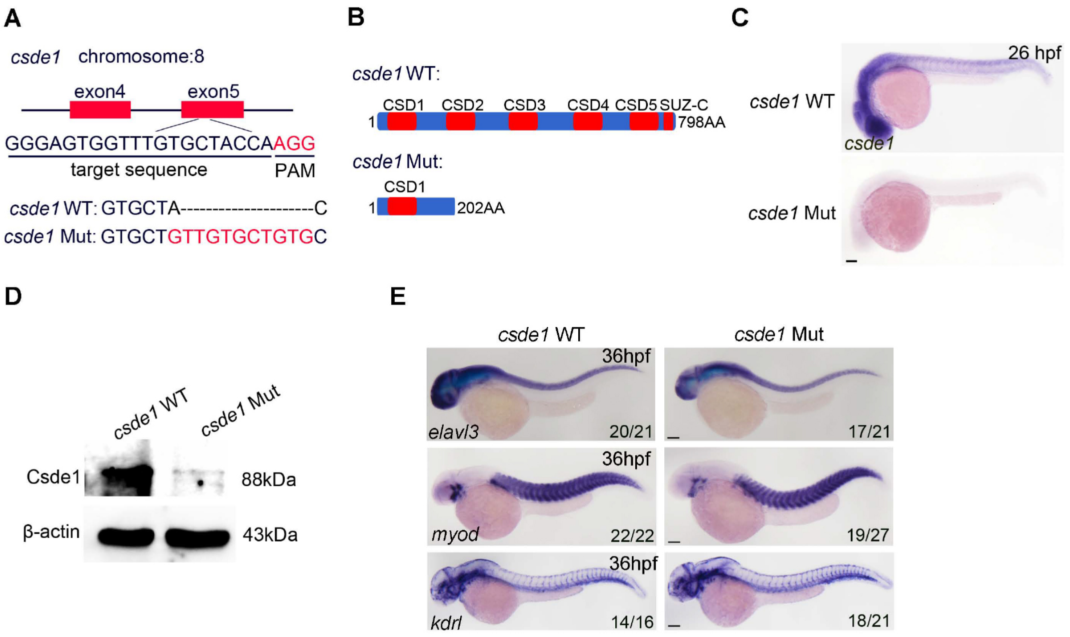

Fig. S3. Generation of *csde1* mutants and general development is not impaired in *csde1* mutants.

(A) The graphical representation of the target site in the fifth exon of *csde1* used for the design of mutants through a CRISPR/Cas9 strategy. The bottom panel showed the - 1+11 bps in the *csde1* target sites.

(B) The predicted truncated Csde1 protein in mutant.

(C) The expression of *csde1* in WT and *csde1* mutants at 26 hpf by WISH. Scale bars, 100 µm.

(D) The protein level of Csde1 was not detectable in *csde1* mutants at 30 hpf.

(E) WISH analysis showing the expression of nervous system markers *elavl3* (arrowheads), somite marker *myod* (arrowheads), and endothelia cells marker *kdrl* in WT and csde1 mutants at 36 hpf. Scale bars, 100 µm. n≥3 replicates. Numbers indicate the number of embryos with respective phenotype/ total number of embryos analyzed in each experiment (E).

Development • Supplementary information

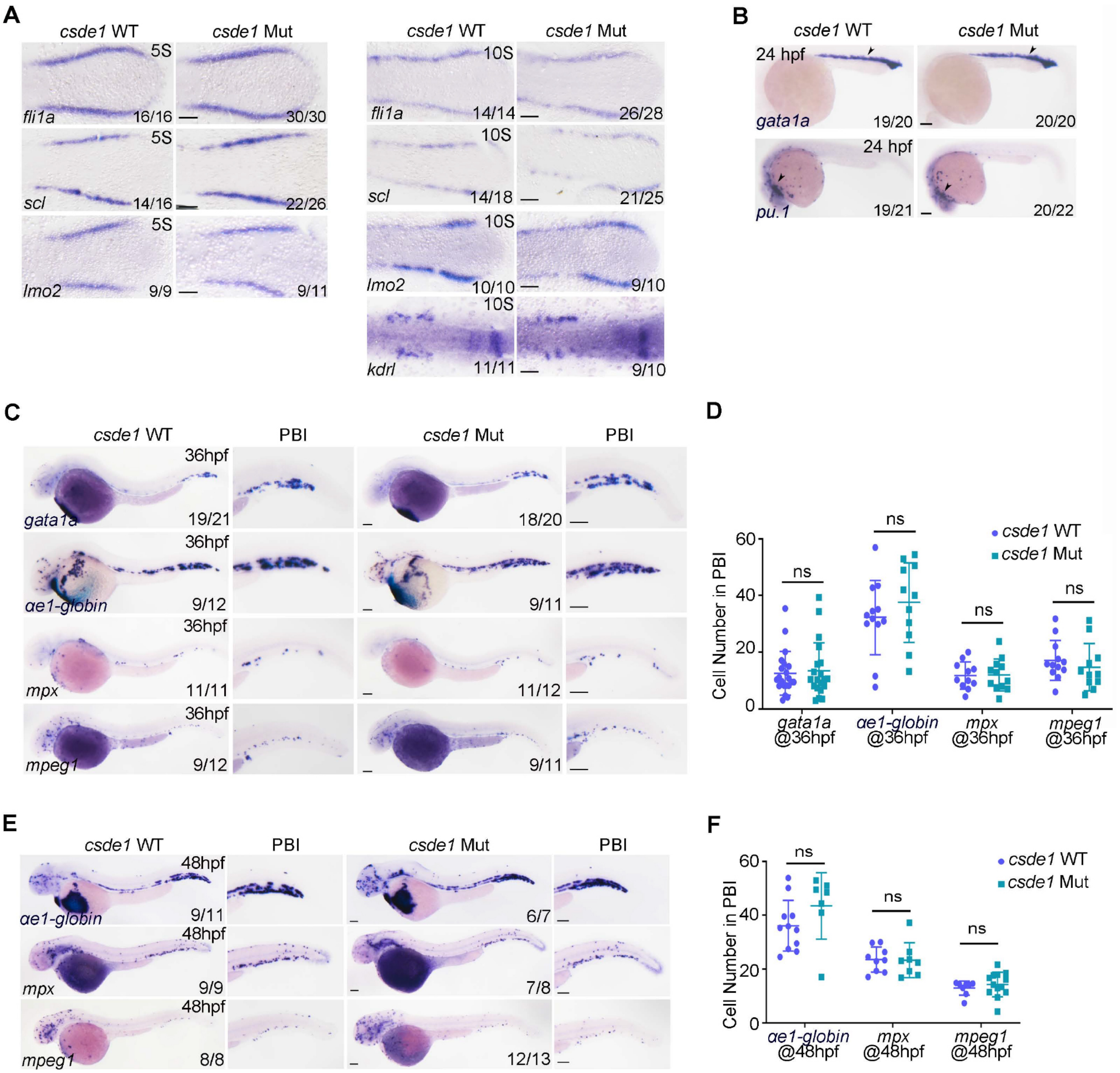
A
csde1 WT
csde1 Mut
5S
fli1a
16/16
30/30
scl
14/16
22/26
lmo2
9/9
9/11
10S
fli1a
14/14
26/28
scl
14/18
21/25
lmo2
10/10
9/10
kdrl
11/11
9/10
B
24 hpf
gata1a
19/20
20/20
pu.1
19/21
20/22
C
PBI
36hpf
gata1a
19/21
18/20
αe1-globin
9/12
9/11
mpx
11/11
11/12
mpeg1
9/12
9/11
D
Cell Number in PBI
ns
gata1a @36hpf
αe1-globin @36hpf
mpx @36hpf
mpeg1 @36hpf
E
48hpf
αe1-globin
9/11
6/7
mpx
9/9
7/8
mpeg1
8/8
12/13
F
αe1-globin @48hpf
mpx @48hpf
mpeg1 @48hpf

Fig. S4. Pre-hematopoietic mesoderm, primitive hematopoiesis and EMP-derived hematopoietic cells were unaltered in *csde1* mutants.

(A) WISH analysis showing the expression of *fli1a*, *scl*, *lmo2* and *kdrl* in WT and *csde1* mutants at 5- and 10-somite stage, respectively. Scale bars, 100 µm.

(B) WISH analysis showing the expression of primitive erythroid marker *gata1a* (arrowheads) and primitive myeloid marker *pu.1* (arrowheads), in WT and *csde1* mutants at 24 hpf. Scale bars, 100 µm.

(C-D) WISH analysis (C) and quantification (D) showing the expression of erythroid marker *gata1a*, *αe1-globin* and myeloid marker *mpx*, *mpeg1*, in WT and *csde1* mutants at 36 hpf. Scale bars, 100 µm.

(E-F) WISH analysis (E) and quantification (F) showing the expression of erythroid marker *αe1-globin* and myeloid marker *mpx*, *mpeg1*, in WT and *csde1* mutants at 48 hpf. Scale bars, 100 µm. PBI, posterior blood island. Error bars, mean ± s.d. Student's *t*-test. ns, not significant. n≥3 replicates. Numbers indicate the number of embryos with respective phenotype/ total number of embryos analyzed in each experiment (A, B, C, E).

Development • Supplementary information

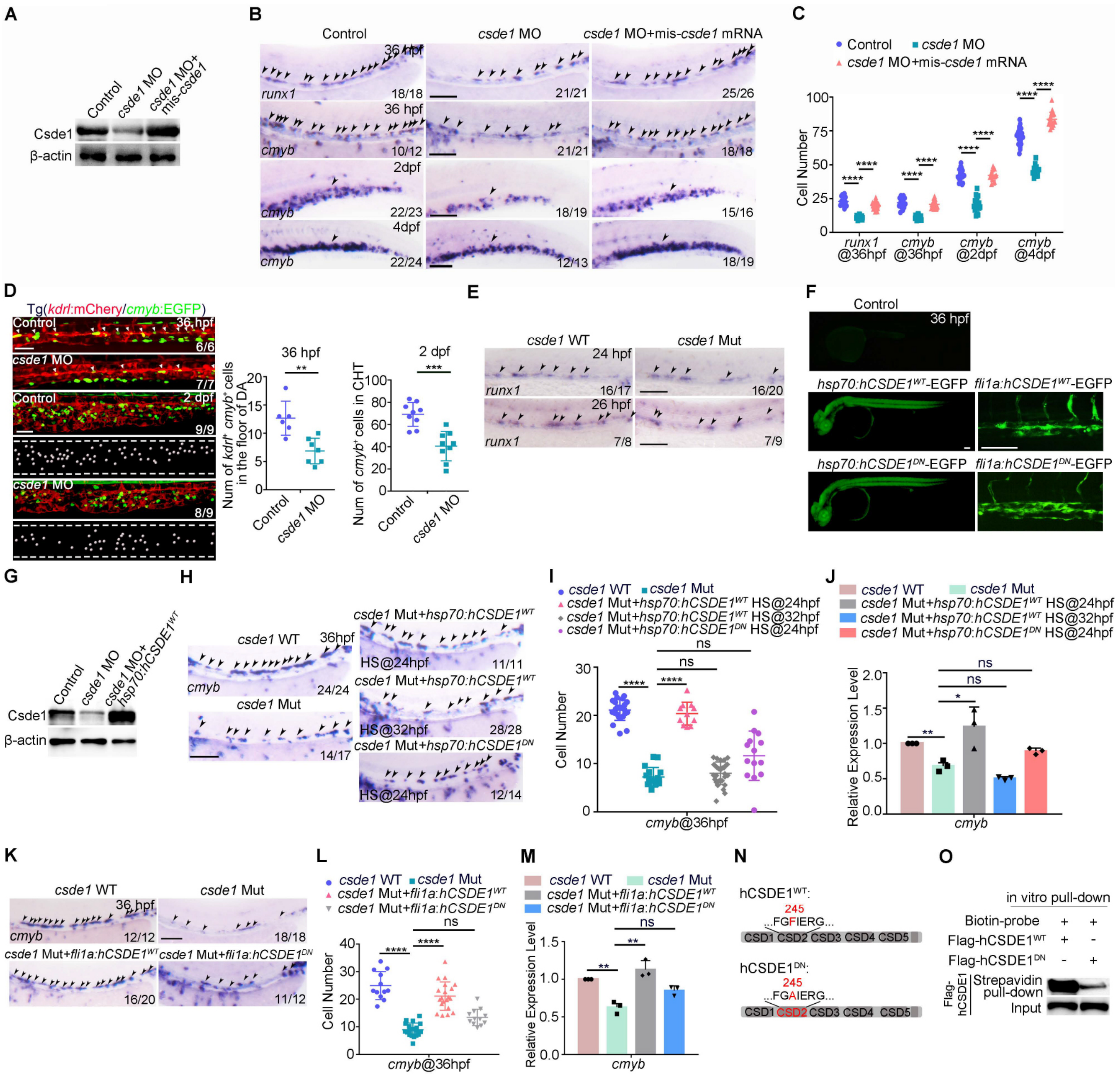

Development • Supplementary information

Fig. S5. Csde1 is required for definitive hematopoiesis.

(A) The protein level of Csde1 in control, *csde1* morphants, and embryos co-injected with *csde1 atg*MO and mis-*csde1* mRNA at 30 hpf.

(B-C) WISH analysis (B) and quantification (C) showing the expression of *runx1* at 36 hpf and *cmyb* (arrowheads) at 36 hpf, 2dpf and 4 dpf were rescued by mis-*csde1* mRNA, compared with *csde1* morphants. Scale bars, 100 µm.

(D) Confocal imaging with quantification (right panel) showing the decreased number of *kdrl*$^{+}$*cmyb*$^{+}$ HECs (white arrowheads) in the AGM region at 36 hpf and *cmyb*$^{+}$ HSPCs in the CHT region at 2 dpf in *csde1* morphants, compared with control group. Scale bars, 50 µm. (E) WISH showing *runx1* expression in *csde1* mutants and WT at 24, and 26 hpf.

(F) The EGFP expression in embryos injected with *hsp70*: *flag-hCSDE1*^{WT}-EGFP, *hsp70*: *flag-hCSDE1*^{DN}-EGFP, *fli1a*: *flag-hCSDE1*^{WT}-EGFP or *fli1a*: *flag-hCSDE1*^{DN}-EGFP at 36 hpf, respectively. Heat shock was performed at 24 hpf. Scale bars, 100 µm. (G) The expression of Csde1 was rescued in embryos injected with *hsp70*: *flag-hCSDE1*^{WT}-EGFP constructs at 30 hpf.

(H) WISH results showing the expression of *cmyb* (arrowheads) in WT, *csde1* mutants, and *csde1* mutants injected with *hsp70*: *flag-hCSDE1*^{WT}-EGFP or *hsp70*: *flag-hCSDE1*^{DN}-EGFP constructs at 36 hpf. Scale bars, 100 µm.

(I) The quantification of the WISH data in H.

(J) qPCR analysis of *cmyb* in WT, *csde1* mutants, and *csde1* mutants injected with *hsp70*: *flag-hCSDE1*^{WT}-EGFP or *hsp70*: *flag-hCSDE1*^{DN}-EGFP constructs at 36 hpf.

(K) WISH results showing the expression of *cmyb* (arrowheads) in WT, *csde1* mutants, and *csde1* mutants injected with *fli1a*: *flag-hCSDE1*^{WT}-EGFP or *fli1a*: *flag-hCSDE1*^{DN}-EGFP constructs at 36 hpf. Scale bars, 100 µm.

(L) Statistical analysis of the WISH in K.

(M) qPCR analysis of *cmyb* in WT, *csde1* mutants, and *csde1* mutants injected with *fli1a*: *flag-hCSDE1*^{WT}-EGFP or *fli1a*: *flag-hCSDE1*^{DN}-EGFP constructs at 36 hpf.

(N) The amino acid of human CSDE1 in red was mutated to generate mutant CSDE1 protein *hCSDE1*^{DN}.

(O) Western blotting showing the Flag-hCSDE1 protein from *Flag-hCSDE1*^{WT} or *Flag-hCSDE1*^{DN}-transfected HEK293 cells pulled down with biotin-labeled probe. Error bars, mean ± s.d. Student's t-test. ns, not significant. *$p < 0.05$, **$p < 0.01$, ***$p < 0.001$, ****$p < 0.0001$. n≥3 replicates. Numbers indicate the number of embryos with respective phenotype/ total number of embryos analyzed in each experiment (B, D, H, K).

Development • Supplementary information

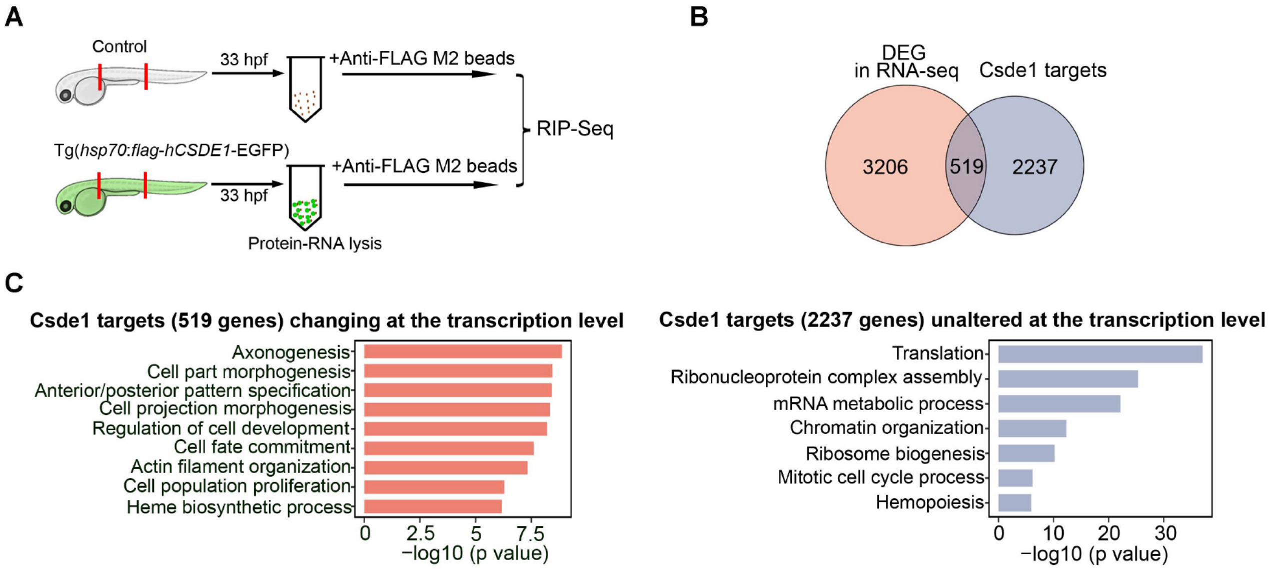

Fig. S6. Identification of Csde1 regulating targets by RNA-seq and RIP-seq.

(A) Schematic representation of RIP-seq experiments using the AGM region of control or Tg(*hsp70*: *flag-hCSDE1*-EGFP) embryos at 33 hpf.

(B) Venn diagram displaying the overlap of differentially expressed genes upon *csde1* deficiency by RNA-seq and Csde1 targets by RIP-seq.

(C) GO analysis of 519 *csde1* target genes regulated at the transcriptional level (left panel) and 2,237 *csde1* target genes regulated at the post-transcriptional level (right panel).

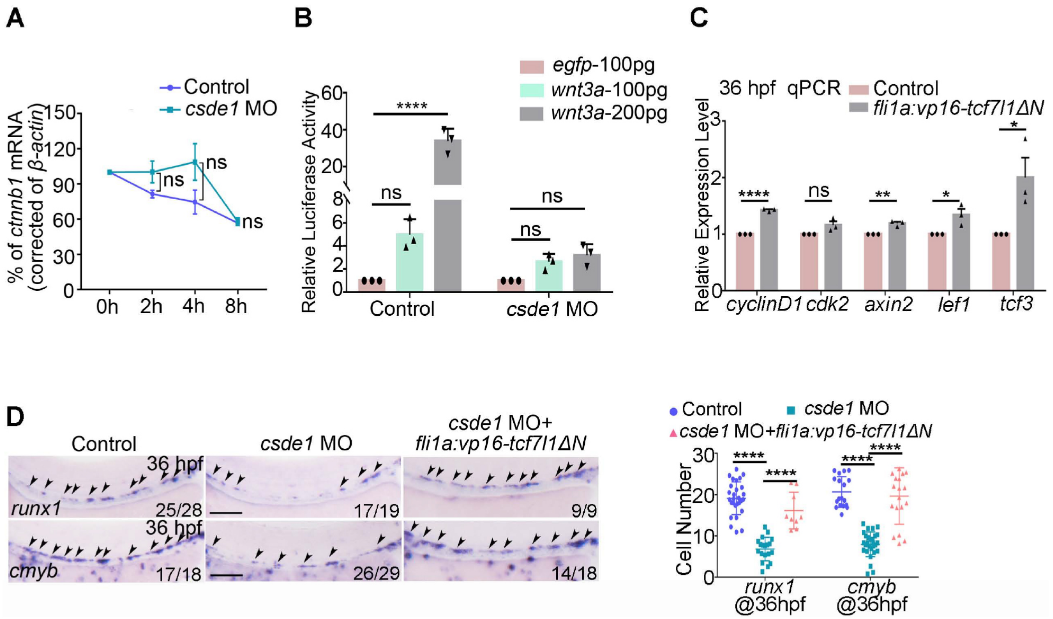

Fig. S7. Csde1 regulates Wnt signaling to affect HSPCs generation.

(A) qPCR analysis of the *ctnnb1* mRNA level at 0 h, 2 h, 4 h, and 8 h after injection of α-amanitin in control and *csde1* morphants.

(B) TOPFlash luciferase reporter assays in *wnt3a* mRNA injected zebrafish embryos with or without *csde1 atg*MO injection.

(C) qPCR analysis showing the increased expression of Wnt targets in embryos injected with *fli1a*:*vp16-tcf7l1ΔN*-tdTomato constructs at 36 hpf.

(D)WISH results showing that endothelial-derived *vp16-tcf7l1ΔN*-tdTomato overexpression rescued the expression of *runx1* and *cmyb* (arrowheads) in the AGM region, compared with *csde1* morphants at 36 hpf, with quantification. Scale bars, 100 µm. Error bars, mean ± s.d. Student's *t*-test. ns, not significant. *$p < 0.05$, **$p < 0.01$, ****$p < 0.0001$. n≥3 replicates. Numbers indicate the number of embryos with respective phenotype/ total number of embryos analyzed in each experiment (E).

Table S1. The primers for plasmid construction, genotyping and qPCR.

Available for download at
https://journals.biologists.com/dev/article-lookup/doi/10.1242/dev.201890#supplementary-data

Table S2. The sequencing metrics for RNA-seq data.

Available for download at
https://journals.biologists.com/dev/article-lookup/doi/10.1242/dev.201890#supplementary-data

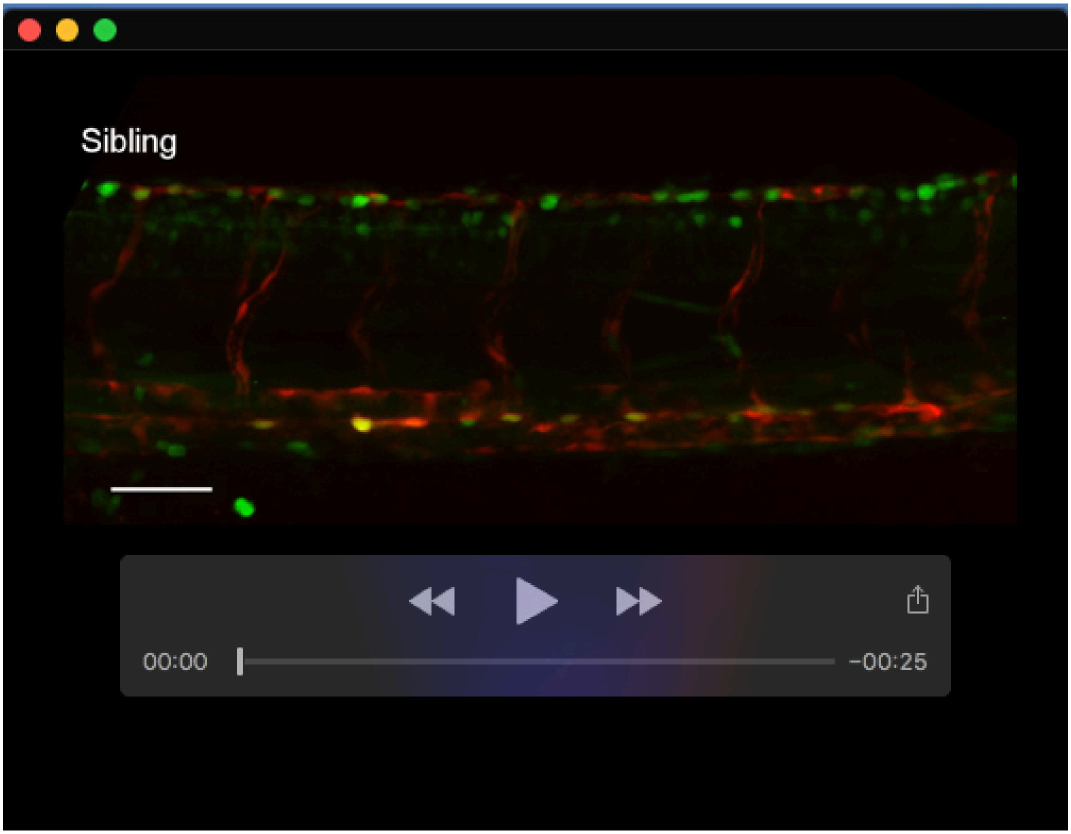

Movie 1. Time-lapse lineage tracing of EHT in control embryos.
The fate transition via EHT was observed in control Tg(*kdrl*:mCherry/*runx1*:en-GFP) embryo from 36 hpf to 41 hpf by Andor Dragonfly 505 confocal microscope. The white arrows indicate the HEC and emerging HSPCs.

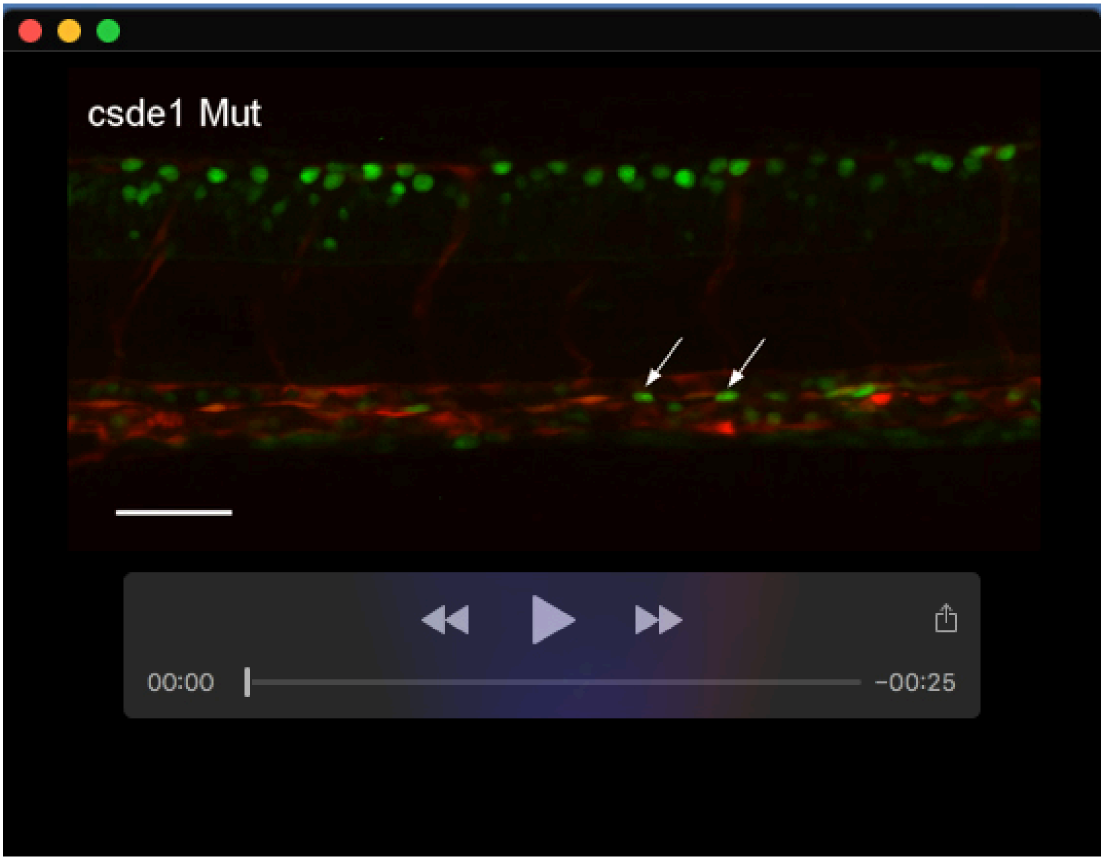

Movie 2. Time-lapse lineage tracing of EHT in *csde1* mutants.

The fate transition via EHT was impaired in *csde1* knockout Tg(*kdrl*:mCherry/*runx1*:en-GFP) embryo from 36 hpf to 41 hpf. The white arrow indicates the HEC and emerging HSPC.

Development • Supplementary information
