# Supplementary material for: The RNA-binding protein CSDE1 promotes hematopoietic stem and progenitor cell generation via translational control of Wnt signaling
Source: Development. 2023 Oct 24;150(21):dev201890. doi: 10.1242/dev.201890 (PMC10652045; doi:10.1242/dev.201890)
Supplement: Supplementary file 1 [file develop-150-201890_review_history.pdf]

Development

# The RNA-binding protein CSDE1 promotes hematopoietic stem and progenitor cell generation via translational control of Wnt signaling

Ying Li, Can Li, Mengyao Liu, Shicheng Liu, Feng Liu and Lu Wang
DOI: 10.1242/dev.201890

**Editor**: Hanna Mikkola

**Review timeline**

| | |
|---|---|
| Original submission: | 17 April 2023 |
| Editorial decision: | 30 May 2023 |
| First revision received: | 21 August 2023 |
| Editorial decision: | 18 September 2023 |
| Second revision received: | 19 September 2023 |
| Accepted: | 19 September 2023 |


---

**Original submission**

First decision letter

MS ID#: DEVELOP/2023/201890

MS TITLE: The RNA-Binding Protein CSDE1 Promotes Hematopoietic Stem and Progenitor Cell Generation via Translational Control of Wnt Signaling

AUTHORS: Ying Li, Can Li, Mengyao Liu, Shicheng Liu, Feng Liu, and Lu WANG

I have now received all the referees' reports on the above manuscript, and have reached a decision. The referees' comments are appended below, or you can access them online: please go to BenchPress and click on the 'Manuscripts with Decisions' queue in the Author Area.

As you will see, the referees express considerable interest in your work, but have some significant criticisms and recommend a substantial revision of your manuscript before we can consider publication. If you are able to revise the manuscript along the lines suggested, which may involve further experiments, I will be happy receive a revised version of the manuscript. Your revised paper will be re-reviewed by one or more of the original referees, and acceptance of your manuscript will depend on your addressing satisfactorily the reviewers' major concerns. Please also note that Development will normally permit only one round of major revision. If it would be helpful, you are welcome to contact us to discuss your revision in greater detail. Please send us a point-by-point response indicating your plans for addressing the referee's comments, and we will look over this and provide further guidance.

Please attend to all of the reviewers' comments and ensure that you clearly highlight all changes made in the revised manuscript. Please avoid using 'Tracked changes' in Word files as these are lost in PDF conversion. I should be grateful if you would also provide a point-by-point response detailing how you have dealt with the points raised by the reviewers in the 'Response to Reviewers' box. If you do not agree with any of their criticisms or suggestions please explain clearly why this is so.

© 2023. Published by The Company of Biologists under the terms of the Creative Commons Attribution License (https://creativecommons.org/licenses/by/4.0/).

Reviewer 1

*Advance summary and potential significance to field*

In this manuscript the authors show that loss of csde1 impaired the endothelial to hematopoietic transition which could not be restored by induced overexpression at 24hpf or EC specific expression of hCSDE1 RNA binding mutant but could be restored by the overexpression of WT and human CSDE1. The authors find that many WNT pathway genes are down regulated in the zebrafish mutants. They show that b-catenin, which is not downregulated in the mutants is a direct target of Csde1. Thus, Csde1 promotes HSPC generation via translational control of b-catenin. Indeed, pull down of b-catenin mRNA showed reduced association with the t eIF4a1a protein in the absence of Csde1. Finally, they showed that constitutively active TCF could rescue the phenotype, thus, verifying the implication of Wnt singnaling.

*Comments for the author*

This is a very complete and interesting work. Some minor modifications will make it better:

-Please explain briefly in the text what is transcriptional score.
-Please do qPCR for validating Cdse1 expression
-At 4dpf most lymphoid and erythroid cells do not come from HSPCs. Can the authors comment on this? Maybe EMPs also play a role in the phenotype.
-Since Csde1 affects the genes mostly post-transcriptionally how can the fish have some deregulated genes. Can the authors comment on this.
-Can the authors rescue the interaction of b-catenin mRNA with eIF4a1a if they overexpress Cdse1?
-The authors could mention the number of embryos on the in situ photos.
-Line 140: the authors are using the abbreviation EC for the first time, so they should explain it.

Reviewer 2

*Advance summary and potential significance to field*

Review of Li et al 2023

*Development*
In this manuscript, the authors report a role for Csde1, a putative RNA-binding protein (RBP), in HSPC generation during zebrafish development. Using genetic mutants and morphants they provide evidence that loss of function in csde1 impairs HSPC production. Their experiments support the idea that the mechanism behind this effect is modulation of the Wnt/&[beta]-catenin pathway by Csde1. This work therefore provides new insight into both the genetic regulation of hematopoietic development and how Wnt/&[beta]-catenin signaling is regulated at the posttranscriptional level. Our major concerns involve the question of what stage Csde1 acts cell autonomously within hematopoietic precursors and the strength of the evidence supporting its role in promoting &[beta]-catenin translation. We believe that this manuscript would be of interest to readers of Development and might be suitable for publication with major revisions.

*Comments for the author*

1- Published data supporting a cell-autonomous role for Wnt signaling in HSPC specification from hemogenic endothelium are relatively weak (Ruiz-Herguido et al., 2012; Goessling et al., 2009; Grainger et al., 2016) and feature obscure mechanisms where Wnt signaling activity is often heavily delayed downstream of reception of the signal (e.g. Grainger et al., 2016). A much clearer requirement for canonical Wnt signaling is found in the need for Wnt activity (especially Wnt3 and Wnt8 family ligands) to ventralize/posteriorize lateral plate mesoderm"LPM"(during early/mid-somitogenesis in zebrafish) by turning on expression of cdx genes and key hox genes (esp. hoxb6b and hoxa9a) in order to bestow hematopoietic competence. Defects in this phase of Wnt signaling would be compatible with some or all of the defects the authors observe and should be excluded. They might also be compatible with the late-stage heatshock rescue (24 hpf), since no one has explored how late Wnt-dependent programming of LPM could occur. The authors should show

© 2023. Published by The Company of Biologists under the terms of the Creative Commons Attribution License (https://creativecommons.org/licenses/by/4.0/).

absence of earlier defects in patterning of hematopoietic mesoderm and hemogenic endothelium in the csde1 mutants. They should examine expression of tal1, lmo2, fli1a kdrl in wild type vs. mutants at earlier time points to exclude patterning defects in pre-hematopoietic mesoderm at early/mid-somite stages like 5-10 somites. Particularly since Csde1 is a putative regulator of Wnt signaling, putative targets of Wnt ventralization/posteriorization of lateral plate mesoderm and upstream of definitive hematopoietic specification, especially cdx1, cdx4, hoxa9a, and hoxb6b need to be evaluated at early/mid-somite stages, like 5-10-somites (e.g. Davidson et al., 2003).

2- The authors need to consider:

Fig. S2A: Better csde1 in situ.

Close ups of AGM region.

Better kdrl fluorescence in situs (Fig. S2A, Fig.1C)

Number of embryos per condition and per experiment is needed.

Csde1 developmental timing series including RT-PCR for 1-c., 6 hpf (shield), tailbud, mid-somite, & 24 hpf.
WISH at shield, mid-somite, and 24 hpf.

3- Fig. S3C shows little transcript in endothelium at 26 hpf. The authors' heat shock rescue experiments (Fig. 2A-C, S5B, C) indicate that Csde1 should function at 24 hpf; but there appears to be little or no endothelial expression of csde1 at 26 hpf. Convincing endothelial expression for csde1 at 24 hpf needs to be demonstrated or the mechanism needs to be reevaluated.

4- *Runx1* needs to be evaluated at 24 hpf in the mutants. Mutant expression is shown at 30 hpf (1F) and 36 hpf (1A). These time points are significantly later than the onset of runx1 HSPC expression at 23-24 hpf.

5- Fig. 1A: *rag1* results are not too compelling. Does the histogram (1A bottom right) depict "decreases" that are typical? If so, this presentation exaggerates the effect.

6- The decreased expression of *hbbae1* at 4 dpf is indicative of a defect in primitive or EMP-derived hematopoiesis not HSC-derived hematopoiesis that would be the presumed output of AGM runx1+ and cmyb+ HSPCs. Hbbae1+ cells at this stage come from primitive or EMP precursors.

7- Fig. 1C: dot plot shows "quantitation of runx1+ cells in the CHT" with a range around 40-55 (mean around 48) for WT and 20-48 (mean around 30) for mut, but a quick count of the representative images shows 64 runx1+ cells in WT and 42 for mut. So, count confidences are diminished.

8- Fig. 1 conclusion: "Csde1 is 'indispensable' for HSPC development" is overstated, since apparently there are still numerous HSPCs in the absence of Csde1. (Also the movies do not strongly make the case for fewer HE cells).

9- Fig. S6 clearly implies that &[beta]-catenin regulation has Wnt signaling consequences (i.e. the signaling pool of &[beta]-catenin is affected). Fig. 5C purports to show that &[beta]-cat protein is down in DA. The vast majority of &[beta]-cat in cells is in the non-signaling pool (i.e. plasma membrane); none of what they're showing appears nuclear (i.e. Wnt signaling active). In fact it all mostly looks like it is located at the plasma membrane. This result is particularly worrisome because it both implies that there is little or no active canonical Wnt signaling in the hemogenic endothelium at the time point under consideration, and also that the vast majority of &[beta]-catenin is in the cell-cell junction pool, as would be expected. Typically, researchers interested in demonstrating Wnt-signaling-specific effects on &[beta]-catenin deplete the plasma membrane pool (usually more than 90%), which otherwise obscures effects on protein levels.

10- Fig 5D, E: The EGFP-ctnnb1 reporter assay needs better description. What UTR domains of ctnnb1 are present? What does the construct that encodes the EGFP &[beta]-catenin fusion look

© 2023. Published by The Company of Biologists under the terms of the Creative Commons Attribution License (https://creativecommons.org/licenses/by/4.0/). 

like (what is the relative positioning of the two cistrons? where are the UTRs? etc.)? How was the putative RNA binding region of the ctnnb1 transcript determined and included in the reporter?

11- Text needs to be clarified on p. 5, line 150 to indicate that “very few homozygous mutants could survive to adulthood…” Also, the significance of not having maternal zygotic mutants is not clear since the authors haven’t shown whether csde1 is maternally expressed (see RT-PCR staging series above).

---

**First revision**

Author response to reviewers' comments

**Response to the reviewers’ comments**

We are very grateful to the reviewers for their insightful comments on our manuscript. We have performed a number of critical experiments and analysis as suggested to improve the quality of our manuscript. The detailed point-by-point responses to reviewers’ comments are shown below.

*Reviewer 1 Advance Summary and Potential Significance to Field:*
*In this manuscript the authors show that loss of csde1 impaired the endothelial to hematopoietic transition which could not be restored by induced overexpression at 24hpf or EC specific expression of hCSDE1 RNA binding mutant but could be restored by the overexpression of WT and human CSDE1. The authors find that many WNT pathway genes are down regulated in the zebrafish mutants. They show that b-catenin, which is not downregulated in the mutants is a direct target of Csde1. Thus, Csde1 promotes HSPC generation via translational control of b-catenin. Indeed, pull down of b-catenin mRNA showed reduced association with the t eIF4a1a protein in the absence of Csde1. Finally, they showed that constitutively active TCF could rescue the phenotype, thus, verifying the implication of Wnt singnaling.*

*Reviewer 1 Comments for the Author:*
*This is a very complete and interesting work. Some minor modifications will make it better:*
*-Please explain briefly in the text what is transcriptional score.*

**Response 1:** Thanks for this suggestion. Transcriptional score in original manuscript is the module score that measures the average expression levels of a set of genes and is calculated using the Seurat function AddModuleScore with the default parameters. AddModuleScore is used to characterize the molecular features (Stuart et al., 2019). We have revised the manuscript as “we *first evaluated module scores to measure the transcriptional levels of genes related to post-transcriptional regulatory processes to characterize the molecular features*” (line 116) and added the description in the Methods (line 531-533).

*-Please do qPCR for validating Cdse1 expression*

**Response 2:** Thanks for the suggestion. We have performed qPCR and WISH to validate *csde1* expression using whole embryos from the one-cell stage to 36 hpf, and found that *csde1* initially displayed maternal expression and later on was highly expressed in the AGM region from 24 to 36 hpf (see **revised Fig.S2B**). Besides, we also confirmed that *csde1* was expressed in ECs using qPCR in sorted *fli1a*:EGFP$^{+}$ ECs (see **revised Fig.S2C**).

© 2023. Published by The Company of Biologists under the terms of the Creative Commons Attribution License (https://creativecommons.org/licenses/by/4.0/).

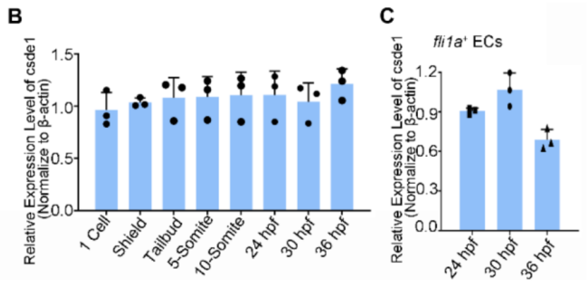

-*At 4dpf most lymphoid and erythroid cells do not come from HSPCs. Can the authors comment on this? Maybe EMPs also play a role in the phenotype.*

**Response 3:** Thanks for this thoughtful comment. To investigate whether *csde1* deficiency affects EMPs, we have performed live imaging in Tg (*cmyb*:EGFP;*gata1*:dsRed) and found that the numbers of EMP (*cmyb*$^+$ *gata1*$^+$) at 36 hpf and 48 hpf in *csde1* mutants were comparable with that in wild-type siblings (see below **Response Figure. 1A**). Furthermore, we have also examined EMP- derived hematopoietic cells, i.e. erythroid (labeled by *gata1a* and *ae1-globin*) and myeloid cells (labeled by *mpx* and *mpeg1*) at 36 and 48 hpf. WISH results showed that EMP-derived hematopoietic cells were unaltered in the *csde1* mutants, compared with wild-type siblings (see below **Response Figure. 1B-E** and **revised Fig. S4C-F**). Thus, these data supported that loss of Csde1 did not affect EMPs. Besides, we re-examined lymphoid and erythroid cells at 5 dpf and the results of WISH and qPCR showed a significant decrease in *csde1* mutants as seen in the **revised Fig. 1A**, **1B**). Together, we speculated that the reduction of lymphoid and erythroid cells in *csde1*- deficiency was mostly due to the defects of HSPCs.

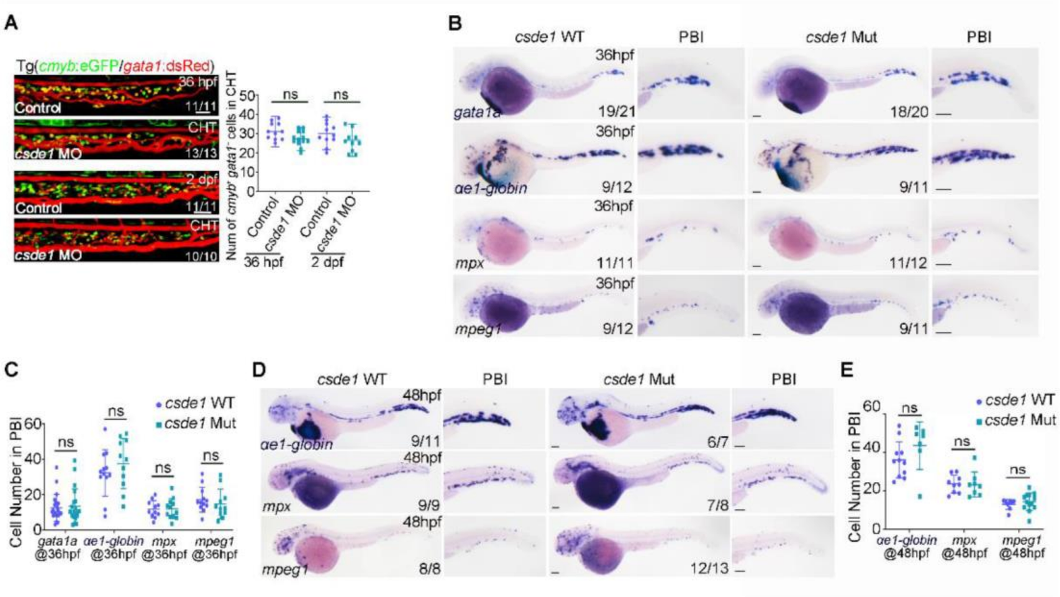

**Response Figure 1. Loss of *csde1* did not affect EMP-derived hematopoietic cells.** (A) Confocal imaging showing *cmyb*+ *gata1*+ EMPs in the CHT region at 36 hpf and 2 dpf in *csde1* morphants and control embryos, with quantification (right panel). Scale bars, 50 μm. CHT, caudal hematopoietic tissue. (B-C) WISH analysis (B) and quantification (C) showing the expression of erythroid marker *gata1a*, *ae1-globin* and myeloid marker *mpx*, *mpeg1*, in WT and *csde1* mutants at 36 hpf. Scale bars, 100 μm. (D-E) WISH analysis (D) and quantification (E) showing the expression of erythroid marker *ae1-globin* and myeloid marker *mpx*, mpeg1, in WT and *csde*1 mutants at 48 hpf. Scale bars, 100 μm. PBI, posterior blood island. Error bars, mean ± s.d. Student’s t-test. ns, not significant. n≥3 replicates. Numbers indicate the number of embryos with respective phenotype/ total number of embryos analyzed in each experiment (A, B, D; right bottom).

-*Since Csde1 affects the genes mostly post-transcriptionally how can the fish have some deregulated genes. Can the authors comment on this.*

© 2023. Published by The Company of Biologists under the terms of the Creative Commons Attribution License (https://creativecommons.org/licenses/by/4.0/).

**Response 4:** Previous studies have demonstrated that CSDE1 controls gene expression mainly through post-transcriptional programs, including regulation of mRNA stability and translation at different stage (*i.e.*, initiation, elongation, or termination) (Guo et al., 2020). For instance, CSDE1 regulates both the stability and translation of *FABP7* mRNA as well as the stability of *VIM* mRNA in hESCs (Ju Lee et al., 2017). CSDE1 could also regulate the levels of mRNAs coding for tumor-promoting and suppressing factors, such as *PTEN* and *SASP* (senescence-associated secretory phenotype) factors (Avolio et al., 2022; Wurth et al., 2016). Additionally, CSDE1 may regulate the expression of some transcripts indirectly through regulating developmental signaling pathways, such as TGF-beta and WNT signaling pathways (Mihailovic et al., 2012), which are downstream targets of CSDE1.
Hence, the observations of the deregulated genes in the *csde1* mutants might be explained by two reasons. Firstly, Csde1 can directly regulate the expression of deregulated genes by controlling their mRNA stability. Secondly, the deregulated genes are downstream genes of some signaling pathways, therefore regulated by Csde1 indirectly.

*-Can the authors rescue the interaction of b-catenin mRNA with eIF4a1a if they overexpress Cdse1?*

**Response 5:** Thanks for this suggestion. We have performed RNA pull down assay and demonstrated that overexpression of *hCSDE1* could restore the attenuated interaction between ß- catenin mRNA with HA-eIF4a1a upon *csde1*-deficiency (see **revised Fig.5H**)

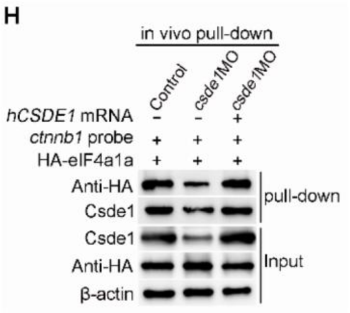


*-The authors could mention the number of embryos on the in situ photos.*

**Response 6:** Thanks for this suggestion. We have added the number of embryos in the revised Figures (see revised Fig. 1A, 1E, 1G; Fig. 2A, 2D; Fig. 6A; Fig. S3E; Fig. S4A, S4B, S4C, S4E; Fig. S5B, S5H, S5K; Fig. S7E).

*-Line 140: the authors are using the abbreviation EC for the first time, so they should explain it.*

**Response 7:** Thanks for pointing out this. We have revised and used "endothelial cells (EC)" for the first time (line 108) in the revised manuscript.

***** *Reviewer 2 Advance Summary and Potential Significance to Field:*

*In this manuscript, the authors report a role for Csde1, a putative RNA-binding protein (RBP), in HSPC generation during zebrafish development. Using genetic mutants and morphants they provide evidence that loss of function in csde1 impairs HSPC production. Their experiments support the idea that the mechanism behind this effect is modulation of the Wnt/&[beta]-catenin pathway by Csde1. This work therefore provides new insight into both the genetic regulation of hematopoietic development and how Wnt/&[beta]-catenin signaling is regulated at the posttranscriptional level. Our major concerns involve the question of what stage Csde1 acts cell autonomously within hematopoietic precursors and the strength of the evidence supporting its role in promoting &[beta]-catenin translation. We believe that this manuscript would be of interest to readers of Development and might be suitable for publication with major revisions.*

© 2023. Published by The Company of Biologists under the terms of the Creative Commons Attribution License (https://creativecommons.org/licenses/by/4.0/).

**Response 8:** We thank the reviewer for the positive and encouraging comments.
(1) Based on our results showing unaffected primitive hematopoiesis and EMP and impaired HEC and HSPC generation upon *csde1* deficiency, we conclude that Csde1 is required for HSPC development, but not primitive hematopoiesis and EMP as we showed in **the revised Fig S4**;
(2) To determine at what stage Csde1 acts cell autonomously in HSPC generation, we have firstly confirmed *csde1* expression in ECs and HSPCs at 24 and 36 hpf using WISH, qPCR and double FISH as shown in the **revised Fig S2**, indicating its potential role in HSPC development. Secondly, we have performed time-course assay for *runx1* expression from 24 hpf to 30hpf (see below **Response Figure. 2)**. WISH showed that the expression of *runx1* at 24 and 26 hpf was slightly reduced in *csde1* mutants, however, at 30hpf *runx1* expression was evidently compromised, indicating Csde1 could affect HSPC development from 24 hpf. Besides, EC-specific- or stage- specific overexpression of human CSDE1 via *fli1a* or *hsp70* promoter could efficiently rescue HSPC defects in *csde1*-deficient embryos, respectively. Thus, the results as shown in **Fig 2** and **revised Fig 5H-M** demonstrated that Csde1 functions in HSPC specification cell autonomously.
Together, these data suggested that Csde1 is required for HSPC specification in a cell-autonomous manner.

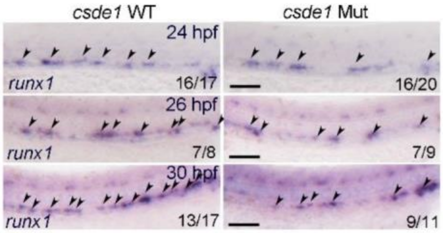


**Response Figure 2. Time-course of *runx1* expression in *csde1* mutants and wild type siblings.** WISH showing *runx1* expression in *csde1* mutants and WT at 24, 26, and 30 hpf. Scale bar, 100μm.

(3) To determine whether and how Csde1 regulates β-catenin, we have firstly demonstrated that the protein level of β-catenin was decreased using western blotting and immunofluorescence, while the mRNA level and stability of *ctnnb1* was unaffected in the absence of *csde1* (see **revised Fig 5A- C** and **Fig S7A)**. Then, an EGFP reporter assay with *ctnnb1* CDS and 3'UTR region containing Csde1-binding sites was performed and the result showed that the EGFP intensity, but not *egfp* mRNA was reduced by loss of *csde1*(see **revised Fig 5D-G)**. Furthermore, based on RNA pull- down assay, we could demonstrate that loss of Csde1 attenuated the interaction between translation initiation factors and *ctnnb1* mRNA, subsequently affected translation of β-catenin (see **revised Fig 5H)**. Together, immunofluorescence, western blotting, reporter assay and pull-down assay together demonstrated that Csde1 could regulate β-catenin translation.

*Reviewer 2 Comments for the Author:*
*1- Published data supporting a cell-autonomous role for Wnt signaling in HSPC specification from hemogenic endothelium are relatively weak (Ruiz-Herguido et al., 2012; Goessling et al., 2009; Grainger et al., 2016) and feature obscure mechanisms where Wnt signaling activity is often heavily delayed downstream of reception of the signal (e.g.Grainger et al., 2016). A much clearer requirement for canonical Wnt signaling is found in the need for Wnt activity (especially Wnt3 and Wnt8 family ligands) to ventralize/posteriorize lateral plate mesoderm"LPM"(during early/mid-somitogenesis in zebrafish) by turning on expression of cdx genes and key hox genes (esp. hoxb6b and hoxa9a) in order to bestow hematopoietic competence. Defects in this phase of Wnt signaling would be compatible with some or all of the defects the authors observe and should be excluded. They might also be compatible with the late-stage heatshock rescue (24 hpf), since no one has explored how late Wnt-dependent programming of LPM could occur. The authors should show absence of earlier defects in patterning of hematopoietic mesoderm and hemogenic endothelium in the csde1 mutants. They should examine expression of tal1, lmo2, fli1a, kdrl in wild type vs. mutants at earlier time points to exclude patterning defects in pre-hematopoietic mesoderm at early/mid- somite stages like 5-10 somites. Particularly since Csde1 is a putative regulator of Wnt signaling, putative targets of Wnt ventralization/posteriorization of lateral plate mesoderm and upstream of definitive hematopoietic specification, especially cdx1, cdx4, hoxa9a, and hoxb6b need to be evaluated at early/mid-somite stages, like 5-10-somites (e.g. Davidson et al., 2003).*

© 2023. Published by The Company of Biologists under the terms of the Creative Commons Attribution License (https://creativecommons.org/licenses/by/4.0/). 

**Response 9:** We thank this reviewer for pointing out this important issue. To examine the early patterning of pre-hematopoietic mesoderm, we have firstly examined *cdx-hox* pathway, including *cdx1*, *cdx4*, *hoxa9a*, and *hoxb6b* at 5- and 10-somite stage. WISH showed that their expression was unaffected by *csde1* deficiency (see below **Response Figure 3A, 3B**), indicating the patterning of posterior lateral plate mesoderm (LPM) was normal in the absence of *csde1*. Given that *cdx-hox* pathway acts to make the posterior LPM competent to respond to genes that specify hematopoietic fate (Davidson et al., 2003), we next detected the expression of *fli1a*, *scl*, *lmo2*, and *kdrl* at 5- and 10-somite stage. And the results clearly showed that the expression of these markers in *csde1* mutants was comparable with that in WT siblings (see below **Response Figure 3C, 3D**), suggesting that Csde1 is not required for pre-hematopoietic mesoderm development. We also examined hemogenic endothelium by detecting *runx1* expression from 24 hpf to 30hpf. WISH showed that the expression of *runx1* at 24 and 26 hpf was slightly reduced, while at 30hpf *runx1* expression was notably decreased in *csde1* mutants, compared with WT siblings (also see **Response Figure 2**).
Together, the patterning of posterior LPM and hematopoietic mesoderm was unaltered, however, the hemogenic endothelium displayed a progressively decrease in *csde1* mutants from 24 hpf, compared with WT siblings.

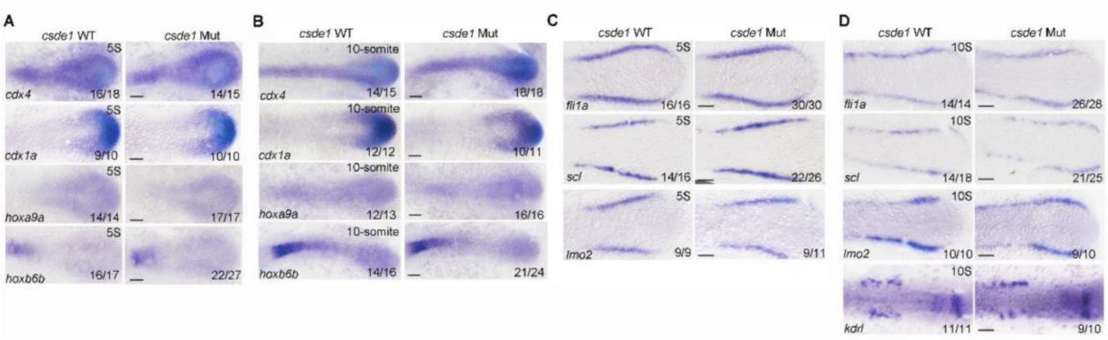


**Response Figure 3. Loss of *csde1* did not affect posterior LPM and hematopoietic mesoderm.** (A-B) WISH analysis showing the expression of *cdx4, cdx1a, hoxa9a*, and *hoxb6b* in WT and *csde1* mutants at 5- and 10-somite stage, respectively. Scale bars, 100 μm. (C-D) WISH analysis showing the expression of *fli1a*, *scl*, *lmo2*, and *kdrl* in WT and *csde1* mutants at 5- and 10-somite stage, respectively. Scale bars, 100 μm.

*2- The authors need to consider:*
*Fig. S2A: Better csde1 in situ. Close ups of AGM region. Better kdrl fluorescence in situs (Fig. S2A, Fig.1C)*

**Response 10:** We thank this reviewer for pointing out this important issue.
(1) We have examined the expression pattern of *csde1* from one-cell stage to 36 hpf. We found that *csde1* displayed maternal expression and was highly expressed in the AGM region from 24 to 36 hpf. Transverse section post WISH at 26 hpf showed the expression of *csde1* in the endothelial cells (see **revised Fig S2A**, inside panel with red and blue circles**)**.
(2) As shown in revised **Fig. S2D and Fig. 1C**, we have showed *kdrl* fluorescence images more clearly in double fluorescence in situ hybridization and live imaging.

*Number of embryos per condition and per experiment is needed.*

Thanks for this suggestion. In the original version, we have performed quantification using ImageJ and statistical analysis of the WISH and live imaging results. In order to more clearly display the number of embryos, we have added the number of embryos below the WISH figures in the revised figures (see revised Fig 1A, 1C, 1E, 1G; Fig 2A, 2D; Fig 5E; Fig 6A, 6C; Fig S3E; Fig S4A, 4B, 4C, 4E; Fig S5B, S5D, S5H, S5K; Fig S7E).

*Csde1 developmental timing series including RT-PCR for 1-c., 6 hpf (shield), tailbud, mid-somite, & 24 hpf. WISH at shield, mid-somite, and 24 hpf.*

We have performed WISH and qPCR to detect *csde1* expression from one-cell stage to 36 hpf and

© 2023. Published by The Company of Biologists under the terms of the Creative Commons Attribution License (https://creativecommons.org/licenses/by/4.0/). 

found that *csde1* displayed maternal expression and then was highly expressed in the AGM region from 24 to 36 hpf (see **revised Fig. S2A, S2B)**

*3- Fig. S3C shows little transcript in endothelium at 26 hpf. The authors' heat shock rescue experiments (Fig. 2A-C, S5B, C) indicate that Csde1 should function at 24 hpf; but there appears to be little or no endothelial expression of csde1 at 26 hpf. Convincing endothelial expression for csde1 at 24 hpf needs to be demonstrated or the mechanism needs to be reevaluated.*

**Response 11:** Thanks for this suggestion. We have examined *csde1* expression from 24 to 36 hpf in the AGM region using WISH, transverse section, qPCR and double fluorescence in situ hybridization (FISH). Based on the results of WISH and qPCR, we found that *csde1* displayed maternal expression and later on was highly expressed in the AGM region from 24 to 36 hpf (**revised Fig. S2A, B**). Moreover, double FISH and qPCR with sorted *fli1a*:EGFP+ ECs confirmed that *csde1* was expressed in ECs at 24, 30 and 36 hpf (**revised Fig. S2C, D**). Therefore, we demonstrated that *csde1* expression is enriched in ECs but its expression in other tissues is also noticed.

*4- Runx1 needs to be evaluated at 24 hpf in the mutants. Mutant expression is shown at 30 hpf (1F) and 36 hpf (1A). These time points are significantly later than the onset of runx1 HSPC expression at 23-24 hpf.*

**Response 12:** Thanks for this suggestion. We have examined *runx1* expression from 24 hpf to 30hpf. WISH showed that the expression of *runx1* at 24 and 26 hpf was slightly reduced, while at 30hpf *runx1* expression was notably decreased in *csde1* mutants, compared with WT siblings (see **Response Figure. 2**).

*5- Fig. 1A: rag1 results are not too compelling. Does the histogram (1A bottom right) depict "decreases" that are typical? If so, this presentation exaggerates the effect.*

**Response 13**: We apologize that we didn't fully describe the histogram. In the original version, the histogram showed the percentage of embryos with normal or decreased expression of *rag1*. To avoid misunderstanding, we re-quantified the *rag1* expression by counting the area of the positive signal in the revised manuscript (Please see the **revised Fig. 1A**).

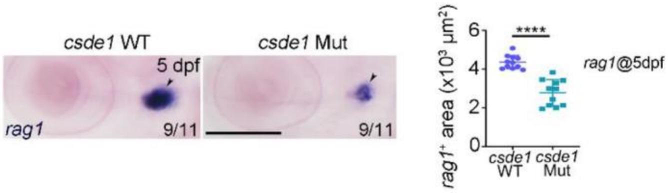


*6- The decreased expression of hbbae1 at 4 dpf is indicative of a defect in primitive or EMP-derived hematopoiesis not HSC-derived hematopoiesis that would be the presumed output of AGM runx1$^+$and cmyb$^+$ HSPCs. Hbbae1$^+$ cells at this stage come from primitive or EMP precursors.*

**Response 14:** Thanks for this suggestion. We have examined erythroid cells at 5 dpf by detecting *gata1a* and *ae1-globin* and the results of WISH and qPCR showed significant decrease in *csde1*-deficient embryos (see **revised Fig. 1A, 1B** and **Response Figure 4**). Besides, we have demonstrated that primitive hematopoiesis and EMP precursors were unaltered in the absence of *csde1* as mentioned above in Response 3 (see **revised Fig. S4** and **Response Figure 1**). Therefore, we reasoned that the decreased expression of *ae1-globin* at 4 dpf in *csde1* mutants was attributable to HSPC defects although the involvement of primitive hematopoiesis cannot be excluded completely.

© 2023. Published by The Company of Biologists under the terms of the Creative Commons Attribution License (https://creativecommons.org/licenses/by/4.0/).

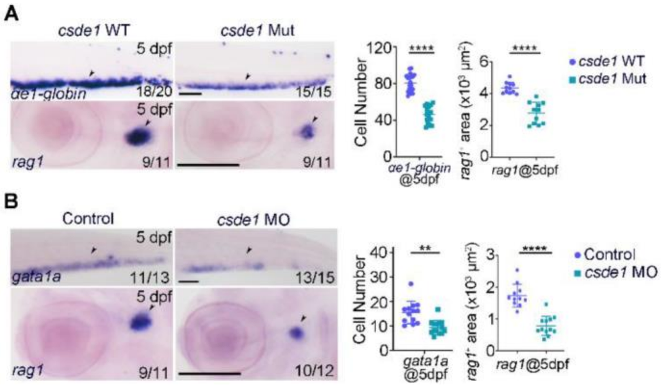


**Response Figure 4. Erythroid and lymphoid lineages were impaired in *csde1*-deficient embryos.** (A-B) Expression of erythroid marker *ae1-globin* or *gata1a* in the CHT region, and lymphoid marker *rag1* (arrowheads) in the thymus region at 5 dpf in *csde1*-deficient and control embryos, with quantification (right panels). Scale bars, 100 μm.

*7- Fig. 1C: dot plot shows "quantitation of runx1$^+$ cells in the CHT" with a range around 40-55 (mean around 48) for WT and 20-48 (mean around 30) for mut, but a quick count of the representative images shows 64 runx1$^+$ cells in WT and 42 for mut. So, count confidences are diminished.*

**Response 15:** We apologize for the inaccurate count. To improve the accuracy of quantitation, we have re-quantified the number of *runx1*$^+$ or *cmyb*$^+$ cells in the CHT using Imaris (Xue et al., 2017), and then have added the analysis in the revised Figures (see **revised Fig 1C, 1D** and **Fig S5D**).

*8- Fig. 1 conclusion: "Csde1 is 'indispensable' for HSPC development" is overstated, since apparently there are still numerous HSPCs in the absence of Csde1. (Also the movies do not strongly make the case for fewer HE cells).*

**Response 16:** Thanks for this suggestion. We have toned down the statement and revised this sentence as "Csde1 is required for HSPC development" (line 178).

*9- Fig. S6 clearly implies that &[beta]-catenin regulation has Wnt signaling consequences (i.e. the signaling pool of &[beta]-catenin is affected). Fig. 5C purports to show that &[beta]-cat protein is down in DA. The vast majority of &[beta]-cat in cells is in the non-signaling pool (i.e. plasma membrane); none of what they're showing appears nuclear (i.e. Wnt signaling active). In fact it all mostly looks like it is located at the plasma membrane. This result is particularly worrisome because it both implies that there is little or no active canonical Wnt signaling in the hemogenic endothelium at the time point under consideration, and also that the vast majority of &[beta]-catenin is in the cell-cell junction pool, as would be expected. Typically, researchers interested in demonstrating Wnt-signaling-specific effects on &[beta]-catenin deplete the plasma membrane pool (usually more than 90%), which otherwise obscures effects on protein levels.*

**Response 17:** Thanks for pointing out this critical point. To detect the expression of β-catenin, we have performed immunofluorescence on transverse section of AGM region in control and *csde1* morphants. By immunofluorescence, we found β-catenin in the cytoplasm of most cells, including ECs, and in the nucleus of few ECs located in the ventral wall of DA (white arrowhead) in control embryos. When *csde1* was knocked-down, the level of β-catenin was obviously reduced in most cells, including ECs, suggesting that Csde1 could affect β-catenin in ECs (see **revised Fig. 5B**). Notably, previous study demonstrated that Wnt/β-catenin activity is restricted to few endothelial nonhematopoietic cells located at the base of the ckit+ hematopoietic clusters in the AGM (Ruiz-Herguido et al., 2012), which is consistent with our findings that β-catenin activity in ECs is required to generate HSCs in the AGM.
Thus, we speculated that loss of Csde1 could affect the level of active-β-catenin in ECs, then impaired HSPC generation.

© 2023. Published by The Company of Biologists under the terms of the Creative Commons Attribution License (https://creativecommons.org/licenses/by/4.0/).

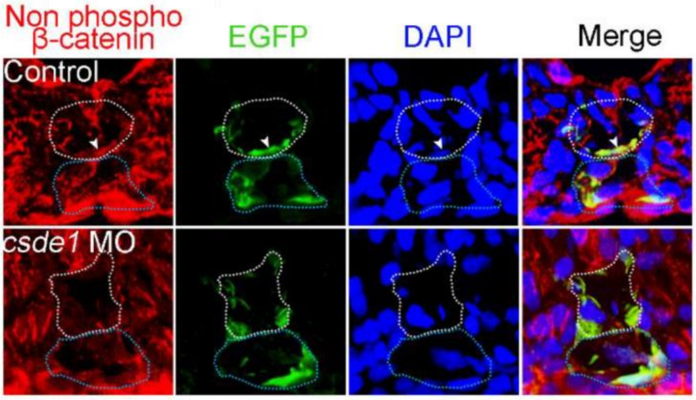


*10- Fig 5D, E: The EGFP-ctnnb1 reporter assay needs better description. What UTR domains of ctnnb1 are present? What does the construct that encodes the EGFP &[beta]-catenin fusion look like (what is the relative positioning of the two cistrons? where are the UTRs? etc.)? How was the putative RNA binding region of the ctnnb1 transcript determined and included in the reporter?*

**Response 18:** Thanks for this suggestion. According to the RIP-seq data as shown in Fig.4B, the putative RNA binding region of *ctnnb1* was located in the CDS and 3'UTR region. And then we cloned the CDS and 3'UTR region of *ctnnb1* and fused it with an EGFP tag to generate *EGFP- ctnnb1* reporter construct (see below **Response Figure 5; Fig. 4B; revised Fig. 5D**). We have revised the description as it writes "*we performed a reporter assay in which the CDS and 3'UTR region of ctnnb1 containing Csde1-binding sites was fused with an EGFP tag. The in vitro transcribed reporter mRNAs were injected into control embryos or csde1 morphants at the one-cell stage and the fluorescence intensity was monitored at the 75% epiboly stage*" (line 304-308) and added an illustration in the **revised Fig. 5D**.

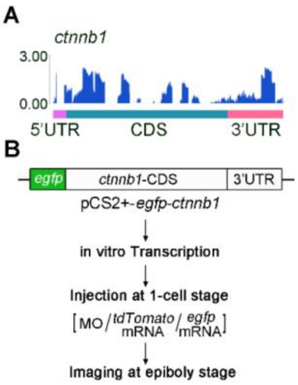


**Response Figure 5. Illustration of the EGFP-*ctnnb1* reporter and experimental procedure for reporter assay.**
(A) RIP-seq reads distribution of *ctnnb1* mRNA compared with control. Blue peaks indicate Csde1 binding sites.
(B) Illustration of the EGFP-*ctnnb1* reporter and experimental procedure for reporter assay.

*11- Text needs to be clarified on p. 5, line 150 to indicate that "very few homozygous mutants could survive to adulthood..." Also, the significance of not having maternal zygotic mutants is not clear since the authors haven't shown whether csde1 is maternally expressed (see RT-PCR staging series above).*

**Response 19:** We have revised the manuscript as it writes "*but only 2% mutants could survive to adulthood and failed to produce maternal-zygotic mutant embryos*". And we also have verified the maternal *csde1* expression using WISH and qPCR as mentioned above in **Response 10**.

© 2023. Published by The Company of Biologists under the terms of the Creative Commons Attribution License (https://creativecommons.org/licenses/by/4.0/).

**References**

Avolio, R., Ingles-Ferrandiz, M., Ciocia, A., Coll, O., Bonnin, S., Guitart, T., Ribo, A., and Gebauer, F. (2022). Coordinated post-transcriptional control of oncogene-induced senescence by UNR/CSDE1. Cell Rep *38*, 110211. Davidson, A.J., Ernst, P., Wang, Y., Dekens, M.P., Kingsley, P.D., Palis, J., Korsmeyer, S.J., Daley, G.Q., and Zon, L.I. (2003). cdx4 mutants fail to specify blood progenitors and can be rescued by multiple hox genes. Nature *425*, 300-306.
Guo, A.X., Cui, J.J., Wang, L.Y., and Yin, J.Y. (2020). The role of CSDE1 in translational reprogramming and human diseases. Cell Commun Signal *18*, 14.
Ju Lee, H., Bartsch, D., Xiao, C., Guerrero, S., Ahuja, G., Schindler, C., Moresco, J.J., Yates, J.R., 3rd, Gebauer, F., Bazzi, H., *et al.* (2017). A post-transcriptional program coordinated by CSDE1 prevents intrinsic neural differentiation of human embryonic stem cells. Nat Commun *8*, 1456.
Mihailovic, M., Wurth, L., Zambelli, F., Abaza, I., Militti, C., Mancuso, F.M., Roma, G., Pavesi, G., and Gebauer, F. (2012). Widespread generation of alternative UTRs contributes to sex-specific RNA binding by UNR. RNA *18*, 53-64.
Ruiz-Herguido, C., Guiu, J., D'Altri, T., Ingles-Esteve, J., Dzierzak, E., Espinosa, L., and Bigas, A. (2012). Hematopoietic stem cell development requires transient Wnt/beta-catenin activity. J Exp Med *209*, 1457-1468.
Stuart, T., Butler, A., Hoffman, P., Hafemeister, C., Papalexi, E., Mauck, W.M., 3rd, Hao, Y., Stoeckius, M., Smibert, P., and Satija, R. (2019). Comprehensive Integration of Single-Cell Data. Cell *177*, 1888-1902 e1821.
Wurth, L., Papasaikas, P., Olmeda, D., Bley, N., Calvo, G.T., Guerrero, S., Cerezo-Wallis, D., Martinez-Useros, J., Garcia-Fernandez, M., Huttelmaier, S., *et al.* (2016). UNR/CSDE1 Drives a Post-transcriptional Program to Promote Melanoma Invasion and Metastasis. Cancer Cell *30*, 694-707.
Xue, Y., Lv, J., Zhang, C., Wang, L., Ma, D., and Liu, F. (2017). The Vascular Niche Regulates Hematopoietic Stem and Progenitor Cell Lodgment and Expansion via klf6a-ccl25b. Dev Cell *42*, 349-362 e344.

---

Second decision letter

MS ID#: DEVELOP/2023/201890

MS TITLE: The RNA-Binding Protein CSDE1 Promotes Hematopoietic Stem and Progenitor Cell Generation via Translational Control of Wnt Signaling

AUTHORS: Ying Li, Can Li, Mengyao Liu, Shicheng Liu, Feng Liu, and Lu WANG

I have now received all the referees reports on the above manuscript, and have reached a decision. The referees' comments are appended below, or you can access them online: please go to BenchPress and click on the 'Manuscripts with Decisions' queue in the Author Area.

The overall evaluation is very positive and we would like to publish a revised manuscript in Development, once the referees' comments have been addressed. As you can see in the Reviewer 3 comments, the reviewer recommends including the highly valuable in situ data that was created in response to Figure 3, as supplementary data.

Reviewer 1

*Advance summary and potential significance to field*

This paper describes the role of the RNA-Binding protein CSDE1 in hematopoietic stem and progenitor cell formation during zebrafish development. Mechanistically this is happening via translational control of Wnt Signaling.

*Comments for the author*

The authors have answered my concerns.

© 2023. Published by The Company of Biologists under the terms of the Creative Commons Attribution License (https://creativecommons.org/licenses/by/4.0/). 

Reviewer 3

*Advance summary and potential significance to field*

This work provides new insight into both the genetic regulation of definitive hematopoietic development and how Wnt/b-catenin signaling is regulated at the posttranscriptional level.

*Comments for the author*

The authors present a revised submission documenting their claim that Csde1 regulates definitive HSPC emergence and Wnt signaling via control of [beta]-catenin stability. The authors were very responsive to reviews and provided extensive rebuttal data that are quite convincing. Our concerns have been addressed and we believe the manuscript would be suitable for publication and of interest to readers of Development with light grammatical editing. I personally think the in situ data presented in Response Figure 3 depicting no alteration in hematopoietic-related gene expression in the lateral plate mesoderm at 5- and 10-somites are extremely informative and would make a great addition in Supplementary Material.

---

**Second revision**

Author response to reviewers' comments

Response to the reviewers' comments

Reviewer 1 Advance summary and potential significance to field
This paper describes the role of the RNA-Binding protein CSDE1 in hematopoietic stem and progenitor cell formation during zebrafish development. Mechanistically this is happening via translational control of Wnt Signaling.

Reviewer 1 Comments for the author
The authors have answered my concerns.

Reviewer 3 Advance summary and potential significance to field
This work provides new insight into both the genetic regulation of definitive hematopoietic development and how Wnt/b-catenin signaling is regulated at the posttranscriptional level.

Reviewer 3 Comments for the author
The authors present a revised submission documenting their claim that Csde1 regulates definitive HSPC emergence and Wnt signaling via control of [beta]-catenin stability. The authors were very responsive to reviews and provided extensive rebuttal data that are quite convincing. Our concerns have been addressed and we believe the manuscript would be suitable for publication and of interest to readers of Development with light grammatical editing. I personally think the in situ data presented in Response Figure 3 depicting no alteration in hematopoietic-related gene expression in the lateral plate mesoderm at 5- and 10-somites are extremely informative and would make a great addition in Supplementary Material.

Response 1: We thank this reviewer for this encouraging evaluation and the suggestion. We have added in situ data of hematopoietic-related genes in the lateral plate mesoderm into Figure S4A.

---

Third decision letter

MS ID#: DEVELOP/2023/201890

MS TITLE: The RNA-Binding Protein CSDE1 Promotes Hematopoietic Stem and Progenitor Cell Generation via Translational Control of Wnt Signaling

© 2023. Published by The Company of Biologists under the terms of the Creative Commons Attribution License (https://creativecommons.org/licenses/by/4.0/).

AUTHORS: Ying Li, Can Li, Mengyao Liu, Shicheng Liu, Feng Liu, and Lu WANG
ARTICLE TYPE: Research Article

I am happy to tell you that your manuscript has been accepted for publication in Development, pending our standard ethics checks.

© 2023. Published by The Company of Biologists under the terms of the Creative Commons Attribution License (https://creativecommons.org/licenses/by/4.0/).
